# Molecular mechanism of the extended oil accumulation phase contributing to the high seed oil content for the genotype of tung tree (*Vernicia fordii*)

**DOI:** 10.1186/s12870-018-1458-3

**Published:** 2018-10-19

**Authors:** Lingling Zhang, Pan Wu, Wenying Lu, Shiyou Lü

**Affiliations:** 1 Key Laboratory of Plant Germplasm Enhancement and Specialty Agriculture, and Wuhan Botanical Garden, Chinese Academy of Sciences, Wuhan, 430074 Hubei China; 2 University of Chinese Academy of Sciences, Beijing, 100049 China; 3 Sino-Africa Joint Research Center, Chinese Academy of Sciences, Wuhan, 430074 China

**Keywords:** Oil content, Fatty acid synthesis, TAG assembly, Tung tree (*Vernicia fordii*), RNA-Seq

## Abstract

**Background:**

Oil from seeds of the tung tree (*Vernicia fordii)* has unique drying properties that are industrially important. We found that the extended oil accumulation period was related to the high seed oil content at maturity among tung tree population. In order to understand the molecular mechanism underlying the high oil content in tung tree seed, Tree H and L were adopted for the further investigation, with seed oil content of about 70 and 45%, respectively. We compared the transcriptomic changes of seed at various times during oil accumulation between the two trees.

**Results:**

Transcriptomes analysis revealed that many genes involved in glycolysis, fatty acid synthesis, and tri-acyl glyceride assembly still kept high expression in the late period of seed oil accumulation for Tree H only. Many genes in fatty acid degradation pathway were largely up regulated in the late period of seed oil accumulation for Tree L only. Four transcription factors related to fatty acid biosynthesis had different expression pattern in the seed oil accumulation period for the two trees. *WRI1* was down regulated and kept the low expression in the late period of seed oil accumulation for the two trees. *PII*, *LEC1* and *LEC1-LIKE* extended the high expression in the late period of seed oil accumulation in Tree H only.

**Conclusions:**

The continued accumulation of oil in the late period of seed oil accumulation for Tree H was associated with relatively high expression of the relevant genes in glycolysis, fatty acid synthesis and tri-acyl glyceride assembly. *PII*, *LEC1,* and *LEC1-LIKE* rather than *WRI1* should play an important role in the oil continual accumulation in the late period of seed oil accumulation in Tree H. This study provides novel insight into the variation in seed oil content and informs plant breeding strategies to maximize oil yield.

**Electronic supplementary material:**

The online version of this article (10.1186/s12870-018-1458-3) contains supplementary material, which is available to authorized users.

## Background

Plant oils have important industrial applications. One important consideration in the exploitation of seed oil for industrial purposes is the oil content, defined as the percentage of oil weight in the total weight of dry seed. Seed oil content can be improved by altering the expression levels of individual genes that encode enzymes involved in oil metabolism [1–4]. Transcription factors (TFs) are reasonable alternatives to single enzyme strategies for overcoming flux bottlenecks associated with multiple enzymatic steps. The WRINKLED1 (WRI1) is identified as a master TF that regulates both glycolysis and fatty acid synthesis by binding to the promoter regions of essential genes in these two pathways [5–7]. Besides, three TFs, LEC1 (leafy-cotyledon protein 1), LEC1-Like (related to LEC1) and PII (P-II homolog) also regulate fatty acid synthesis [8, 9]. Over-expression of *WRI1* gene could stimulate expression of genes that encoded enzymes involving in fatty acid biosynthesis [6, 7, 10], but did not consistently improve the oil content in the target tissue of a variety of transgenic plants [4, 6, 10–12]. An additional concern was that gene overexpression, even if it improved the oil content, may have deleterious effects, for example reducing seed number [13]. It would seem that we have incomplete understanding of the orchestration of the relevant plant metabolic pathways that confer high seed oil content.

In an oil bearing plant species population, high oil content was reportedly correlated with high expression of the biosynthetic genes coding for enzymes in the fatty acid and tri-acyl glyceride (TAG) pathways; this has been noted for castor bean (*Ricinus communis L.*) [14, 15], oil palm (*Elaeis guineensis Jacq.*) [16], *Jatropha curcas* [17, 18], and sesame (*Sesamum indicum L.*) [19]. Oil content of plant organs has been evaluated in a variety of plant species, from herbal to woody plants [20]. For instance, oil palm accumulated about 90% oil in its mesocarp [21], tung tree seed about 50% [22], and Arabidopsis seed about 30% [4]. The variance in oil content among these oil bearing species reflects that characteristics of oil content are complex. The oil bearing plants with high oil content may possess various molecular mechanisms to finally result in the considerable accumulation of oil in the oil bearing organ. Thus, the variance in oil content among genotypes may provide a shortcut to discover some molecular mechanisms for high oil content.

Tung tree, *Vernicia fordii*, is a northern subtropical deciduous woody species in the Euphorbiaceae family that is native to China [23]. It is a cross-pollination plant species with about 6 months for tung tree fruit to mature after fertilization. Tung oil begins to rapid accumulation about 15 to 16 weeks after the flowering. The matured time varied among different genotypes, but focused in the late September to early October in Wuhan, Hubei Province, China. Tung tree mainly distributes in Sichuan, Hubei, Guizhou, and Hunan provinces, and the Chongqing municipality, the main production base of tung oil in the history of China [24, 25]. Lots of local varieties were developed in the history of China, but were basically disappeared with the cutting or natural death because of the appearance of tung oil substitute products since the late 1980s. Nowadays, tung trees are basically in the semi-wild status. Population genetic analysis showed that, currently, tung tree germplasm in China had a relatively high genetic diversity [25].

The endosperm of tung tree seed is rich in oil, and oil content is about 50% [22]. Tung oil is one of the highest quality plant drying oils available, an important feedstock for industrial goods and biodiesel [26–28]. About 80% of the fatty acid components in the oil are α-eleostearic acid (α-ESA or 9 *cis*, 11 *trans*, and 13 *trans* octadecatrienoic acid) [27, 28]. This fatty acid imparts the industrially important drying qualities to the oil. Tung tree germplasm nursery was established at Wuhan, China in 2008, from where the plant materials were acquired for this study. In previous work with tung tree, we constructed a genomic DNA isolation method [29], developed microsatellite markers [24, 30], and evaluated the genetic diversity of our collection [25]. We also collected data on tung oil yield, which revealed substantial diversity: oil content ranged from 40 to 70% among individuals. In this study, twelve genotypes of tung tree, representative of the low (about 40%), middle (about 50–60%) and high oil contents (about 70%), were selected from the population to measure oil rate accumulation curve. We noted that high oil content was often associated with a long period of oil accumulation, compared to the genotypes with low oil content. Using the comparative transcriptome method, we detected this alternative molecular mechanism for high oil content in the endosperm of tung tree. This study will provide new strategies for genetic improvement and metabolic engineering program of oil plants.

## Methods

### Tung tree germplasm

Our lab established a tung tree germplasm nursery at Wuhan, Hubei, China in 2008, which included 527 individuals from 12 provinces of China, covering Sichuan, Hubei, Hunan, Guizhou, Shaanxi, Anhui, Zhejiang, Fujian, Guangdong, Guangxi, Jiangxi province and Chongqing independent municipalities. Based on the data of oil content of tung tree population, twelve individuals, representative of the low (about 40%), middle (about 50–60%) and high (about 70%) oil contents were selected from this nursery to measure the time course of oil accumulation. Tung tree was a cross-pollination plant, and based on our data in this study (Additional file 1: Figure S1), it seemed that oil content for the different fruits during the late period of oil accumulation was basically consistent, indicating that pollen may have little effect on the oil content. Tung fruits were first harvested 14 weeks (July 13, 2016) after flowering (April 10, 2016), and thereafter subsequent samples were taken every one to 2 weeks until the fruit mature (around October 1, 2016). In order to eliminate the probable effect of environment on oil accumulation, we only chose the fruits from the branches in the middle of the trunk at the similar height. One fruit was taken at each period from each genotype. Each fruit contained 4–6 seeds, 2–3 seeds as one biological repeat, and the rest 2–3 as another biological repeat. The endosperms from the 2–3 seeds for each biological repeat were mixed together, then immediately frozen in liquid nitrogen, and ground into powder in liquid nitrogen in the lab. The powders for each biological repeat were divided into two copies: one for oil content measurement and the other for RNA isolation.

### Oil content measurement

The seed endosperm powder was freeze-dried at − 80 °C for 30 h, at which point almost all the water was gone. Because the protocol for measuring oil content was laborious and expensive, the powders from the two replicates were mixed together to get one average sample. Twenty mg of dried powder were used for oil content measurement. Fatty acid methyl esters (FAMEs) were prepared using potassium methoxide transesterification, and extracted using hexane. Butylated hydroxytoluene was included as an antioxidant in FAMEs reaction and extraction at a final concentration of 0.01% (*w*/*v*). FAMEs were separated and quantified by gas chromatography (GC, Agilent 7820A). GC analyses were performed as described by Dyer et al. [31].

### Plant materials for RNA sequencing

**Fig. 1:**
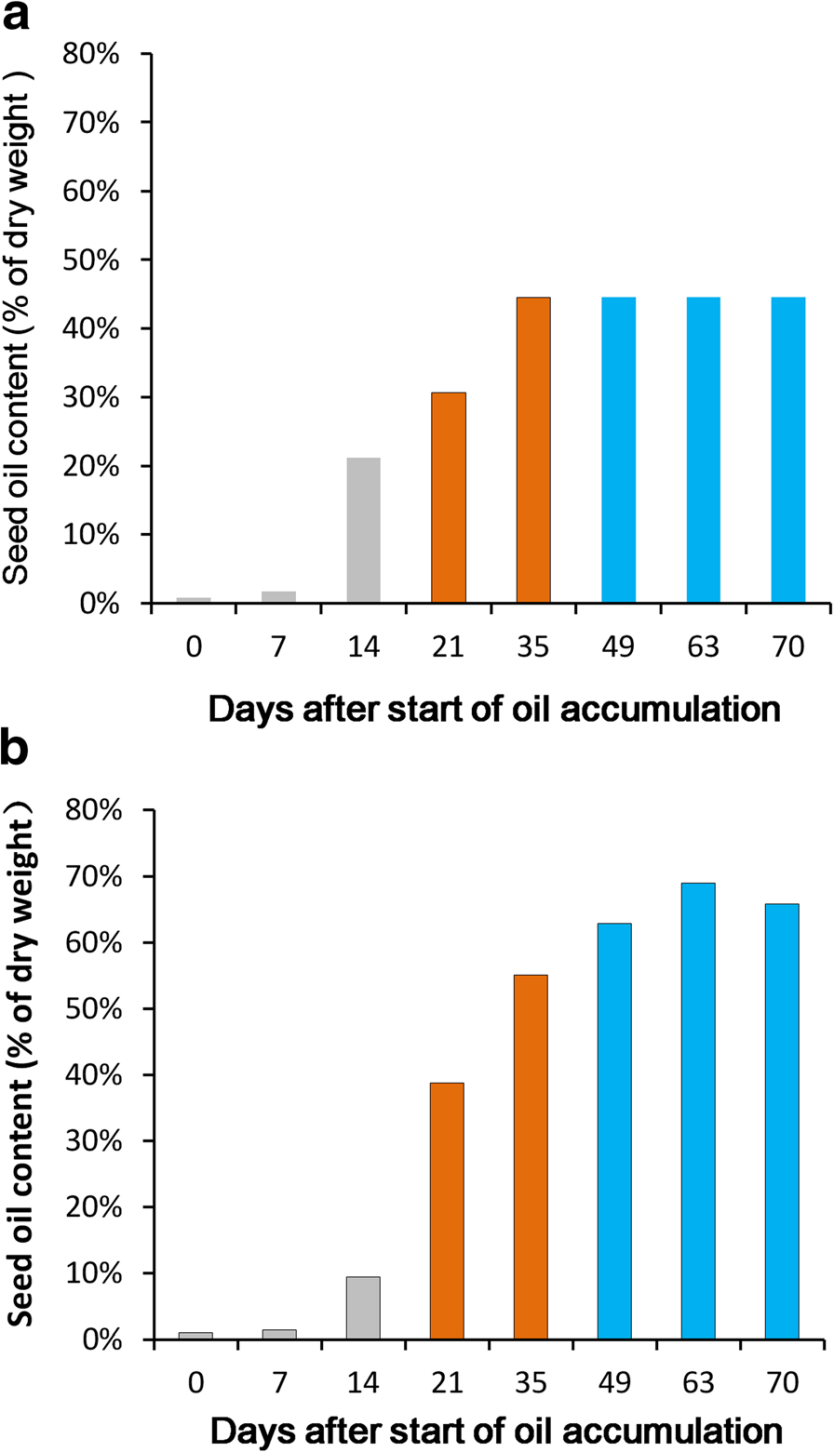
Time course of tung oil accumulation in tung tree seed. **a** Tree L, with seeds that were low in oil; **b** Tree H, with seeds that were high in oil. Three oil accumulation periods were defined: early, from Day 0 to 14 (gray color); middle, from Day 15 to 35 (orange color); and late, from Day 36 to 70 (blue color)

From the twelve initial samples, we selected two with the biggest difference in oil content for RNA sequencing, based on the oil content of the mature endosperm: Tree H had highest oil content (about 70%) and Tree L had lowest oil content (about 45%). We used the time course of oil accumulation that had been measured earlier to define the start point of oil accumulation, Day 0. As shown in Fig. 1, the point, 2 weeks before the obvious increase (hundred times increase compared with the background value of about 0.1%) in oil accumulation was defined as Day 0 of oil accumulation. Seven sampling times were adopted in the further analysis: 7, 14, 21, 35, 49, 63, and 70 days after the start point of oil accumulation.

### RNA isolation, cDNA library preparation, and sequencing

RNA was extracted from tung seeds from Trees H and L at five time points after the start point of oil accumulation: Day 7, 14, 21, 35, and 49 (with two replicates, for a total of 20 samples) using an RNA extraction kit (BioTeck, Beijing, China). Samples were treated with RNase-free DNase (BioTeck, Beijing, China) using on-column DNase digestion. RNA quality detection, cDNA synthesis and library preparation were according to the methods of Chen et al. [32]. The cDNA library was sequenced on an Illumina iSeq™ 2500 platform and 150 bp single-end reads were generated. Raw reads were processed by removing reads containing adapter, reads containing ploy-N and low-quality reads, the clean data (clean reads) were obtained. At the same time, Q20, Q30 and GC-content were calculated.

### Transcriptomic analysis

Transcriptomic analysis in this study was carried out at BMKCloud platform (https://www.biocloud .net/). Tung tree genome has been sequenced by our lab [33]. The clean reads were mapped to Tung tree genome with Tophat2 tools software [34]. The number of mapped clean reads for each gene was counted and normalized using the DESeq package [35]. Gene expression levels were calculated as reads per kilobase of exon model per million mapped reads (FPKM). Gene function was annotated based on the following databases: Nr (NCBI non-redundant protein sequences); Nt (NCBI non-redundant nucleotide sequences); Pfam (Protein family); KOG/COG (Clusters of Orthologous Groups of proteins); Swiss-Prot (A manually annotated and reviewed protein sequence database); KO (KEGG Ortholog database); GO (Gene Ontology) [36, 37]. DEGseq was used to identify DEGs, and DESeq software was for the screen of significant DEGs [35, 38]. DESeq provided statistical routines for determining differential expression in digital gene expression data using a model based on the negative binomial distribution. The corrected *P*-values from this method accounting for multiple tests were used as the key factor, which was the false discovery rate (FDR). FDR < 0.01 and ∣log2 (fold change) ∣ > 1 were set as the thresholds for DEGs. Oil content was no more increased after Day 35 for Tree L, but was still increased at Day 49 for Tree H. In order to detect the molecular mechanism of the extended oil accumulation phase in Tree H that contributed to its high seed oil content, we made four RNA comparisons at the two sampling times of Day 35 and Day 49 (Fig. 1): Tree H at 35 days vs. Tree H at 49 days (H35 vs. H49), L35 vs. L49, L35 vs. H35 and L49 vs. H49. As a control, another three comparisons were made: Comparison L7 vs. H7, L14 vs. H14, and L21 vs. H21. Functional annotation of DEGs was performed to test for enrichments in KEGG pathway using KOBAS software [39]. A significance threshold of 0.01 was used for term identification in KEGG enrichment analysis.

### Quantitative RT-PCR analysis

A total of 1 μg of RNAs was used to synthesize cDNA using PrimeScript™RT Kit (Cat. RR047A, TaKaRa, Japan). The cDNA was diluted ten times, and then used as a template. The reaction solution contained SYBR® PremixExTaq™ II (Tli RNaseH Plus) (Cat. RR820A, TaKaRa, Japan) and was conducted in an ABI 7500 FAST Real Time PCR Detection System (Applied Biosystems, US). Quantitative primers for validation of DEGs are listed in Additional file 2: Table S6. Due to the stable expression in the ten samples of tung tree seed in this study (Additional file 3: Figure S7), *60s ribosomal protein L18a* (*Rp119A*, Tung tree ID tung.gene.scaffold67.00040) was used as the internal control to normalize the relative amount of mRNAs for all the samples. The relative expression levels of the selected genes, normalized to the tung tree *Rp119A*, were calculated using the 2-ΔCt method. All reactions were performed with three replicates.

## Results

### Time course of oil accumulation in twelve tung trees

We defined the period with the apparent increase for oil content in tung tree seed as rapid oil accumulation. The time course of oil rapid accumulation in the seeds of 12 selected trees varied, as did their final oil content (Additional file 1: Figure S1). In general, we noted that trees with seeds that were about 70% oil content in the mature endosperm had a comparably longer rapid oil accumulation period of 5 to 7 weeks (Additional file 1: Figure S1c), whereas trees with seeds that had about 50% oil content had a shorter rapid oil accumulation period of 2 to 4 weeks (Additional file 1: Figure S1a). Trees with seeds that had about 60% oil content had a middle rapid oil accumulation period of 4 to 6 weeks, except one only with 2 weeks of oil quick accumulation (Additional file 1: Figure S1b). We concluded that the extended period of accumulation should be associated with the high seed oil content at maturity.

To examine the molecular mechanisms of high oil accumulation in tung tree seed, we selected two tung tree specimens that varied in the amount of oil in the mature endosperm and conducted a comparative transcriptome analysis. Tree H had about 70% oil rate in mature endosperm, while Tree L had about 45%. Tung oil began to rapid accumulate 15–16 weeks after pollination in Wuhan, Hubei province, China, which was varied among different genotypes (Additional file 1: Figure S1). We defined three oil accumulation periods: early, from Day 0 to 14; middle, from Day 15 to 35; and late, from Day 36 to 70. As shown in Fig. 1, seed oil rapidly accumulated during the early and middle period both for Tree H and L: at Day 35, the oil content was 55.11 and 44.52%, respectively. However, Day 35 was the end of the oil quick accumulation for seeds from Tree L, while it continued to increase in seeds from Tree H, reaching to about 70% during the late accumulation period. It indicated that oil accumulation in the late period was important for the further improvement of oil content.

### Analysis of differentially expressed genes (DEGs) during accumulation of oil in tung seeds

The cDNA libraries from 20 samples representing five time points during oil accumulation in the seeds of Trees H and L were sequenced using the Illumina HiSeq™2500 platform. An overview of RNA-Seq reads and mapping results to the tung tree genome is presented in Additional file 2: Tables S1 and Table S2, respectively. We made four RNA comparisons: Tree H at 35 days vs. Tree H at 49 days (H35 vs. H49), L35 vs. L49, L35 vs. H35 and L49 vs. H49. A total of 4565 differentially expressed genes (DEGs) were identified in the four comparisons (Additional file 2: Table S3). As a control, another three comparisons were made: Comparison L7 vs. H7, L14 vs. H14, and L21 vs. H21. The number of DEGs was: 1450 for H35 vs. H49 (888 up- and 562 down-regulated), 1181 for L35 vs. L49 (727 up- and 454 down-regulated), 505 for L7 vs. H7 (240 up- and 265 down-regulated), 1459 for L14 vs. H14 (1033 up- and 516 down-regulated), 814 for L21 vs. H21 (413 up- and 401 down-regulated), 850 for L35 vs. H35 (406 up- and 444 down-regulated), and 3306 for L49 vs. H49 (1584 up- and 1722 down-regulated; Additional file 4: Figure S2). Further analysis, it was found that total DEGs number between the two trees was largely increase in the later period of oil accumulation, about 2 to 3 times of that in the early and middle period of oil accumulation. Besides, the number of DEGs associated with Comparisons H35 vs. H49 and L35 vs. L49 were similar, but a Venn diagram revealed that only 196 DEGs (13.52% of the total DEGs in Comparison H35 vs. H49, 16.60% of the total DEGs in Comparison L35 vs. L49) were shared both by these two comparisons.

Further hierarchical clustering was employed to observe the overall expression pattern of the DEGs among the 20 samples. The results were shown in Additional file 5: Figure S3: the blue bands represented low gene expression, and the red bands represented high gene expression. The samples were clustered into two mega paraphyletic groups. Two samples, both from Tree L sampled at 49 days after start point of oil accumulation, (designated L49–1 and L49–2) were placed into one group, while the remaining samples were assigned to another group.

From these results, we suggested that the global gene expression was different during the late period of oil accumulation between the two trees.

### Kyoto encyclopedia of genes and genomes (KEGG) was used to detect enrichment of DEGs

**Fig. 2:**
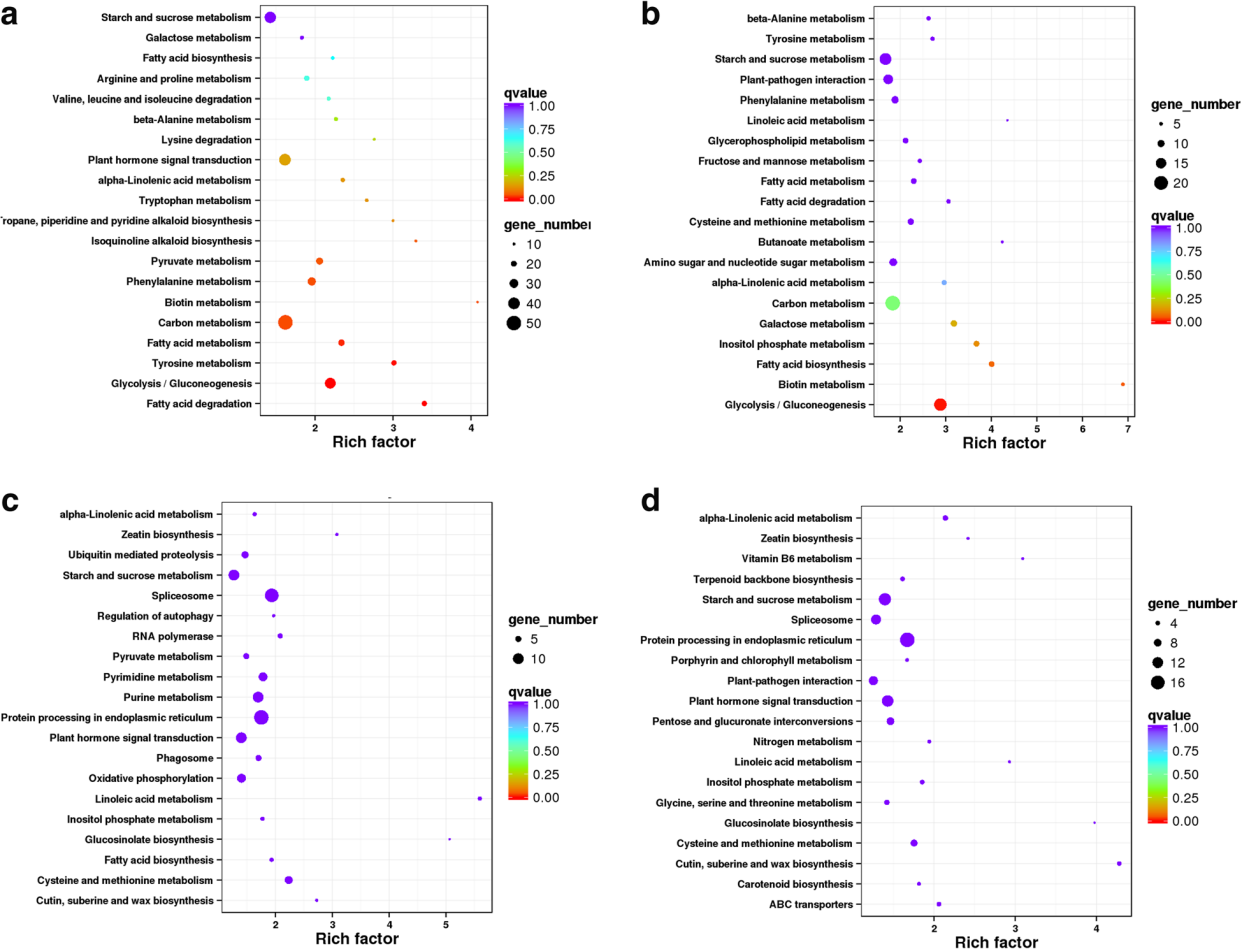
KEGG enrichment analyses for differentially expressed genes (DEGs). **a** Comparison L49 vs. H49; **b** Comparison L35 vs. L49; **c** Comparison L35 vs. H35; and **d** Comparison H35 vs. H49. (L indicated Tree L, with low final oil seed content; H indicated Tree H, with high final seed oil content; numbers following tree indicated days after start point of oil accumulation). Rich factor, enrichment factor, the ratio value of DEGs and all genes devoted in certain pathways; Qvalue, the corrected *P* value in multiple hypothesis testing; Gene_Number, DEGs number enriched in certain pathway

Unigene KEGG annotation [36] was aimed at the DEGs from the comparisons described above. KEGG pathway enrichment analysis of DEGs revealed the enrichment of the “Fatty acid degradation pathway” (*P* = 0.0002), the “Glycolysis/Gluconeogenesis pathway” (*P* = 0.0003), and the “Tyrosine metabolism pathway” (*P* = 0.0013) (Fig. 2a) in the Comparison L49 vs. H49; the “Glycolysis/Gluconeogenesis pathway” (*P* = 0.0040) in the Comparison L35 vs. L49 (Fig. 2b); and “Photosynthesis - antenna proteins” (*P* = 0.000) in the Comparison L14 vs. H14 (Table 1). None of pathways were significantly enriched (*P* < 0.01) in the Comparison L35 vs. H35 (Fig. 2c), Comparison H35 vs. H49 (Fig. 2d), and the rest Comparisons. The details are shown in Additional file 2: Table S4.

**Table 1:** KEGG pathway enrichment of differentially expressed genes (DEGs) in multiple comparisons

	#Pathway	KO	Enrichment Factor	Q-value	GeneNumber
L7 vs. H7	\				
L14 VS. H14	Photosynthesis - antenna proteins	ko00196	12.23	1.E-06	9
L21 VS. H21	\				
L35 VS. H35	\				
L49 VS. H49	Fatty acid degradation	ko00071	3.4	2.E-04	18
	Glycolysis / Gluconeogenesis	ko00010	2.2	3.E-04	37
	Tyrosine metabolism	ko00350	3.01	1.E-03	18
H35 VS. H49	\				
L35 VS. L49	Glycolysis / Gluconeogenesis	ko00010	2.88	4.E-03	18

L, seed from Tree L with low final oil seed content;

H, seed from tree H with high final oil content;

Arabia number following H or L indicated days after start point of oil accumulation;

Pathway, Name for KEGG pathway;

KO, KEGG Orthology;

Enrichment Factor, the ratio value of DEGs and all genes devoted in certain pathways;

Q-value, the corrected P value in multiple hypothesis testing;

Gene Number, DEGs number that enriched in certain pathway;

“**\**”, no pathways that enriched in the pathway

### High expression of the genes involved in glycolysis extended into the late period of oil accumulation in the seeds of tree H

**Fig. 3:**
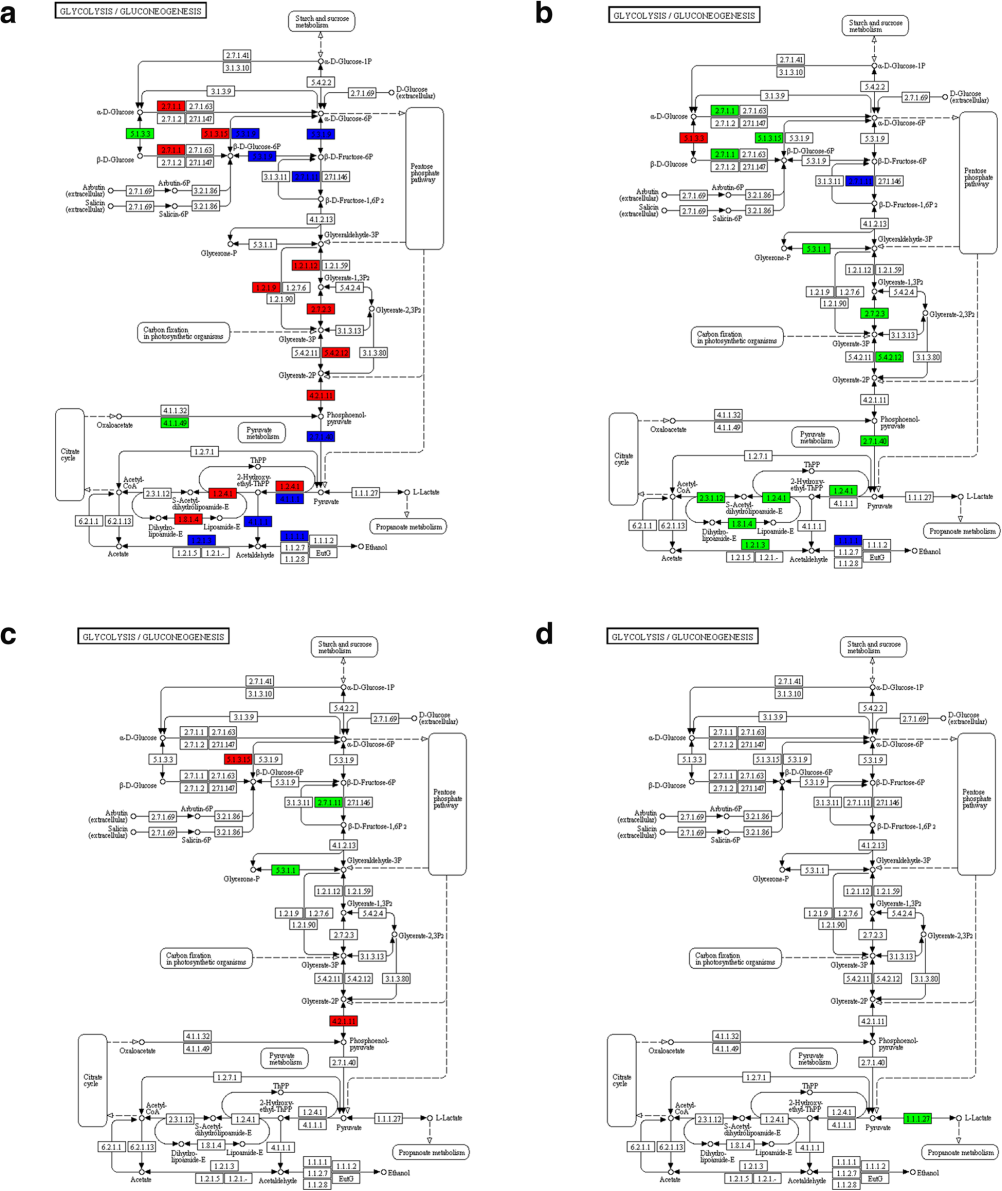
DEGs in glycolytic pathways in tung tree seed. **a** Comparison L49 vs. H49; **b** Comparison L35 vs. L49; **c** Comparison L35 vs. H35; and **d** Comparison H35 vs. H49. The red and green color referred to up- and down-regulated DEGs, respectively. Blue denoted the up and down regulated genes both in certain reactions. (L indicated Tree L, with low final oil seed content; H indicated Tree H, with high final seed oil content; numbers following tree indicated days after start point of oil accumulation)

In higher plants, the main carbon source for storage oil synthesis is sucrose, which could be converted to pyruvate via glycolysis pathway in non-green tissues. Pyruvate is the main precursor for acetyl-CoA molecules that is destined for fatty acid synthesis. In KEGG pathway enrichment analysis of the DEGs, “Glycolysis/Gluoconeogenesis” pathway was enriched both in the Comparisons L49 vs. H49 and L35 vs. L49 (Fig. 2). As shown in Fig. 3, many glycolysis genes were down-regulated in samples L49 compared to samples L35 (Fig. 3b), while gene expression differences between H49 and H35 was minimal (Fig. 3d). Also, many genes were up-regulated in H49 compared to L49 (Fig. 3a), with little difference between H35 and L35 (Fig. 3c).

**Fig. 4:**
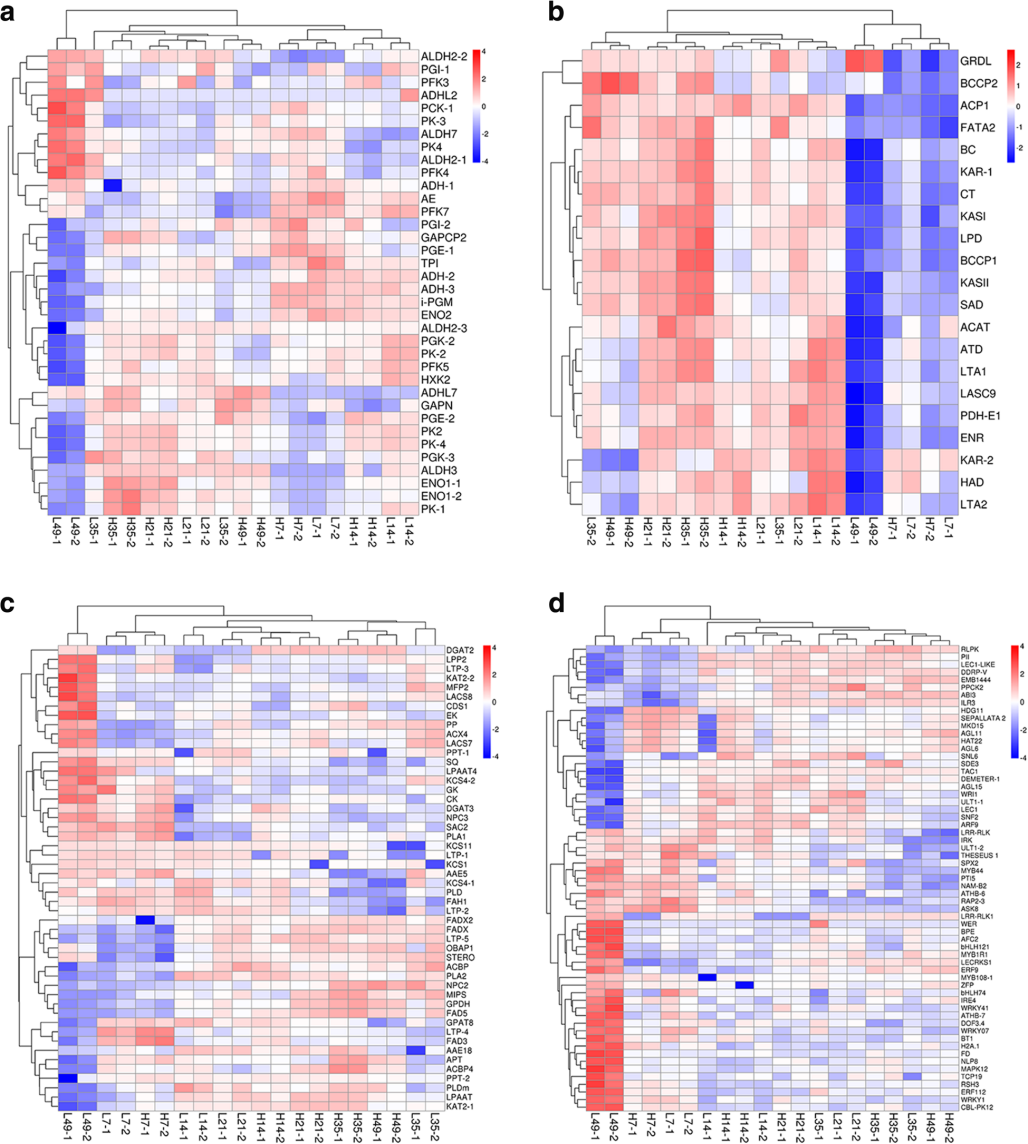
Heatmaps of DEGs from four comparisons among the 20 samples. **a** Glycolysis, **b** Fatty acid synthesis, **c,** Acyl metabolism, **d** Transcription factors (TFs). Four comparisons: L49 vs. H49, L35 vs. L49, L35 vs. H35 and H35 vs. H49. The color referred to fold change (log 10). Fold change of each DEG among the 20 sample was calculated individually. The red and blue color referred to up and down regulation, respectively. Shades change was related to fold change. Notice samples with 49 days after the start point of oil accumulation in Tree L were separated from the other 18 samples in all the four heatmaps except the heatmap for fatty acid synthesis which were clustered together with the samples at 7 days after the start point of oil accumulation from Tree L and H only accumulating a few of oil. The full name of genes and other information in the heatmaps could be found in Additional file 1: Table S5

**Fig. 5:**
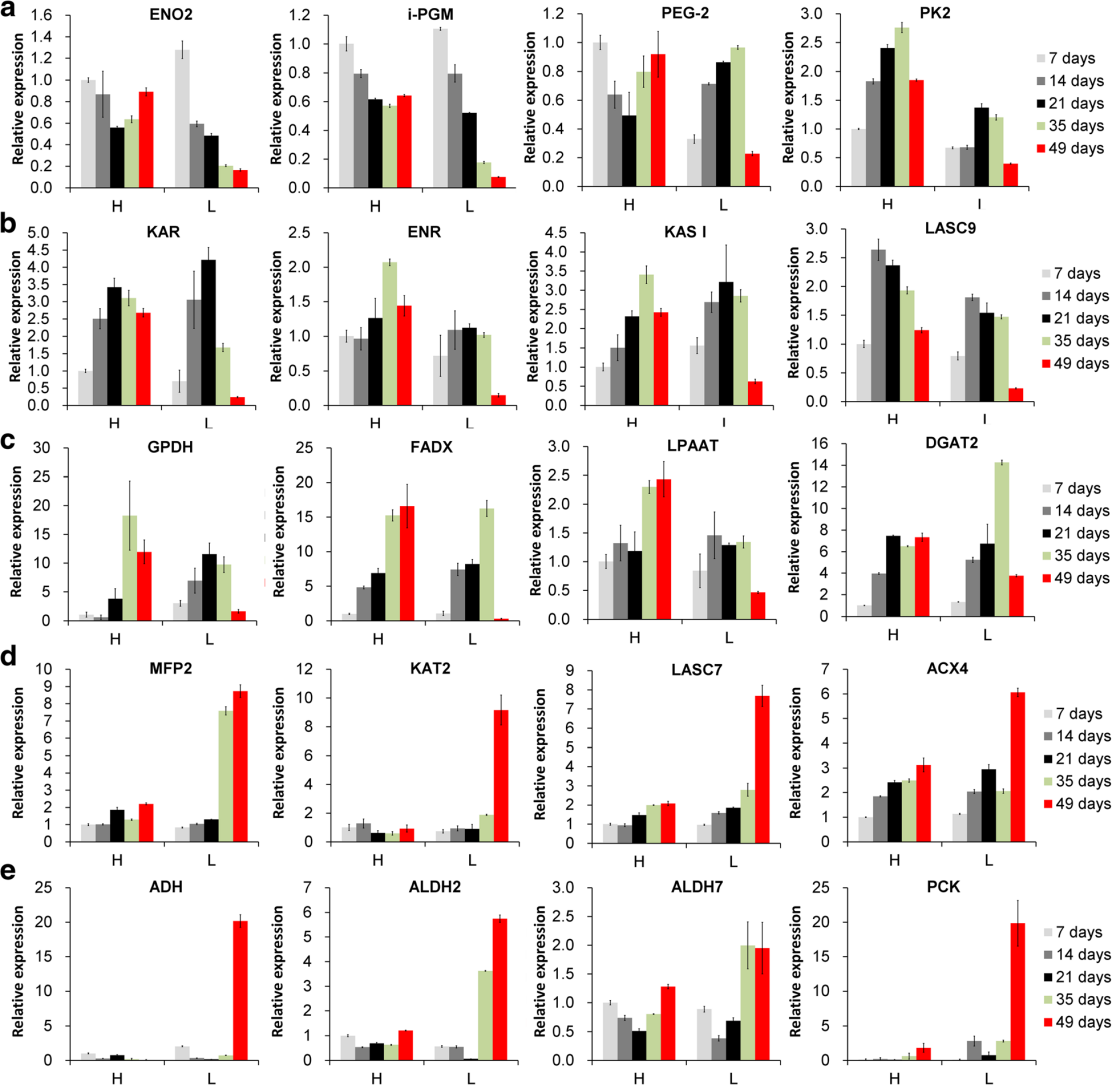
Expression of representative DEGs in multiple pathways by RT-qPCR. The Sample H7 was as a reference sample that was from the seventh day after the start point of oil accumulation in Tree H. **a** Glycolysis; **b** Fatty acid biosynthesis; **c** TAG assembly; **d** Fatty acid degradation; **e** Pyruvate metabolism. L indicated Tree L, with low final oil seed content; H indicated Tree H, with high final seed oil content. Times in legend indicated time after start point of oil accumulation. Abbreviations: FPKM, reads per kilobase of exon model per million mapped reads (FPKM); ENO2, enolase; i-PGM, 2,3-bisphosphoglycerate-independent phosphoglycerate mutase, PEG-2, putative glucose-6-phosphate 1-epimerase; PK2, plastidial pyruvate kinase 2; KAR, ketoacyl-ACP reductase; ENR, enoyl-ACP reductase; KAS I, ketoacyl-ACP synthase I, LASC9, long chain acyl-CoA synthetase 9; GPDH, glycerol-3-phosphate dehydrogenase; DGAT2, type 2 diacylglycerol acyltransferase; LPAA2, 1-acyl-sn-glycerol-3-phosphate acyltransferase 2: FADX, fatty acid conjugase/desaturase; MFP2, MULTIFUNCTIONAL family protein; ACX4, Acyl-coenzyme A oxidase 4;, KAT2, 3-ketoacyl-CoA thiolase 2; LACS7, Long chain acyl-CoA synthetase 7; ADH, alcohol dehydrogenase 1; ALDH2, aldehyde dehydrogenase family 2, ALDH7, aldehyde dehydrogenase family 7; PCK, phosphoenolpyruvate carboxykinase

Further hierarchical clustering was employed to observe the expression pattern of the DEGs that had been annotated to glycolysis in anyone of the public databases at Biocloud platform (https://www.biocloud .net/). As we had predicted, the expression pattern of nearly all the DEGs involved in glycolysis was essentially reversed in the L49 samples compared to all the other samples (Fig. 4a). More than half of genes were up-regulated in the early and middle oil accumulation period for both trees, and still kept relatively high expression in the late accumulation period of Tree H, while the genes expression was low during the late accumulation period of Tree L (samples L49). For instance, four critical genes in glycolysis, *phosphoenolpyruvate enolase* (*ENO*), *phosphoglycerate mutase* (*PGM*), *glucose-6-phosphate 1-epimerase* (*PEG*), and *pyruvate kinase* (*PK*) were highly expressed in the early and middle oil accumulation period of both trees and in the late oil accumulation period of Tree H, but had low expression in the L49 samples (Fig. 5a). We concluded that the continual oil accumulation late in the seed development required relatively high expression of genes in the glycolysis pathway. Figure 4 also displayed ten genes that were highly up regulated in L49 samples in comparison to the rest, including one *phosphoenolpyruvate carboxykinase* (*PCK*), three *aldehyde dehydrogenase* (*ALDH*), two *alcohol dehydrogenase* (*ADH*), and so on. The pyruvate could be used for fatty acid synthesis or transformed into other products, such as aldehydes. It has been noted that ALDH catalyzes the oxidation of aldehydes, when added exogenously or endogenously generated [40]. ADH facilitates the interconversion between alcohols and aldehydes [40]. PCK is the content-limiting step in the metabolic pathway that produces glucose [41]. As shown in Fig. 5e, *ALDH*, *ADH,* and *PCK* were up-regulated in L49 compared to the other samples, indicating pyruvate should be not considerably used for fatty acid synthesis late in oil accumulation in Tree L.

### High expression of genes involved in fatty acid synthesis extended into the late oil accumulation period in tree H

**Fig. 6:**
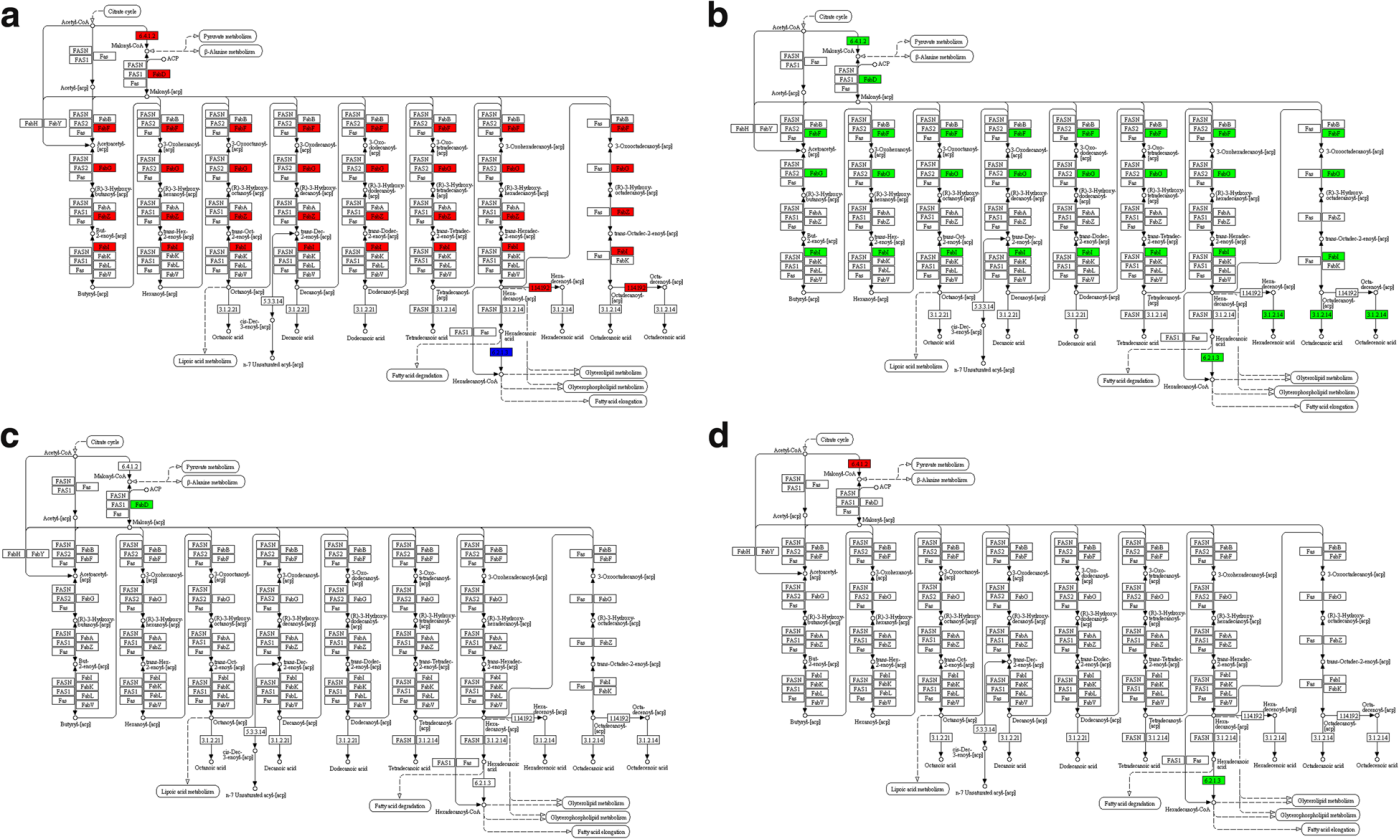
DEGs in fatty acid synthesis pathways. **a** Comparison L49 vs. H49; **b** Comparison L35 vs. L49; **c** Comparison L35 vs. H35; and **d** Comparison H35 vs. H49. Red and green denoted up- and down-regulated genes, respectively. Blue denoted the up and down regulated genes both in certain reactions. L indicated Tree L, with low final oil seed content; H indicated Tree H, with high final seed oil content

The KEGG pathway analysis of DEGs showed that, 49 days after the start point of oil accumulation, many critical genes involved in fatty acid biosynthesis were down-regulated in the seeds from Tree L compared to the seeds from Tree H (Fig. 6a). Many fatty acid biosynthesis genes were also down regulated in 49 days compared to 35 days after the start point of oil accumulation in Tree L (Fig. 6b). The pattern is similar to that which we observed for the DEGs in glycolysis pathways (Fig. 3). Further hierarchical clustering was employed, enabling us to observe expression pattern of DEGs in fatty acid synthesis for all the samples. Figure 4b showed that expression of these genes was low at the seventh day after the start point of oil accumulation in both trees, and increased rapidly during the early and middle stage of oil accumulation. These genes expression was still high in the late period of oil accumulation in Tree H, but declined sharply, to below the level at Day 7, in Tree L. Figure 5b showed the different expression pattern of four DEGs in fatty acid biosynthesis between the two trees, including *Ketoacyl-ACP Reductase* (*KAR*), *Enoyl-ACP Reductase* (*ENR*), *Ketoacyl-ACP Synthase I* (*KAS I*) and *Long chain acyl-CoA synthetase 9* (*LASC9*) [17, 21, 22, 42]. The results were consistent with the suggestion that continued accumulation of oil in the late period of Tree H was associated with high expression of the relevant genes.

### High expression of genes in α-ESA production and TAG assembly extended into the late period of oil accumulation in tree H

Hierarchical clustering was employed to observe expression pattern of DEGs in glycerolipid or glycerophospholipid metabolism. As shown in Fig. 4c, the two L49 samples had quite different expression patterns compared to the rest samples, and we suggested that acyl metabolism was quite different at the late period of oil accumulation in the two trees. *Glycerol-3-phosphate dehydrogenase* (*GPDH*) serves as a major link between carbohydrate metabolism and lipid metabolism, and its product is sn-glycerol 3-phosphate, a component for TAG assembly. In this study, *GPDH* was sharply down-regulated in samples L49 compared to samples H49 and L35, similar to the pattern we observed for the glycolysis and fatty acid synthesis genes (Fig. 5). Nascent oleic acid is synthesized in plastids and transferred to the endoplasmic reticulum, where it is successively catalyzed by tung tree oleate desaturase (encoded by *vfFAD2*) and fatty acid conjugase/desaturase (encoded by *vfFADX*) to form α-ESA [31]. In this study, *FADX* showed similar pattern as *GPDH* (Fig. 5c). Fatty acid could be assembled into TAG by acyl transferase such as glycerol-3-Phosphate (GPAT9) [43], acyltransferase1-acyl-sn-glycerol-3-phosphate acyltransferase 2 (LPAAT) [44] and acyl-CoA: diacylglycerol acyltransferase (DGAT2) [45]. In this study, *DGAT2* also showed similar pattern as *GPDH* and *FADX* (Fig. 5c). DGAT2 from tung tree increased the α-ESA content in Arabidopsis seeds that contained the tung tree *FADX* genes [3, 45]. In this study, two acyl transferases, *LPAAT* (Fig. 5c) and *PLA2* (*Phospholipase A2-alpha*, Additional file 2: Table S5) also showed similar expression patters as *DGAT2* and *FADX*, indicating that they may be involved in α- ESA assembly into TAG as DGAT2. It may be, then, that the extended period of oil in Tree H requires high expression of those genes involved in α-ESA production and TAG assembly.

Other genes involved in glycerolipid or glycerophospholipid metabolism were highly up regulated in L49 comparison to other samples (Additional file 6: Figure S4): this result further suggest a major shift in acyl metabolism in the late period of oil accumulation in the seeds of Tree L that did not occur in the seeds from Tree H.

### Up-regulation of genes in the fatty acid degradation pathway during the late period of oil accumulation in tree L

**Fig. 7:**
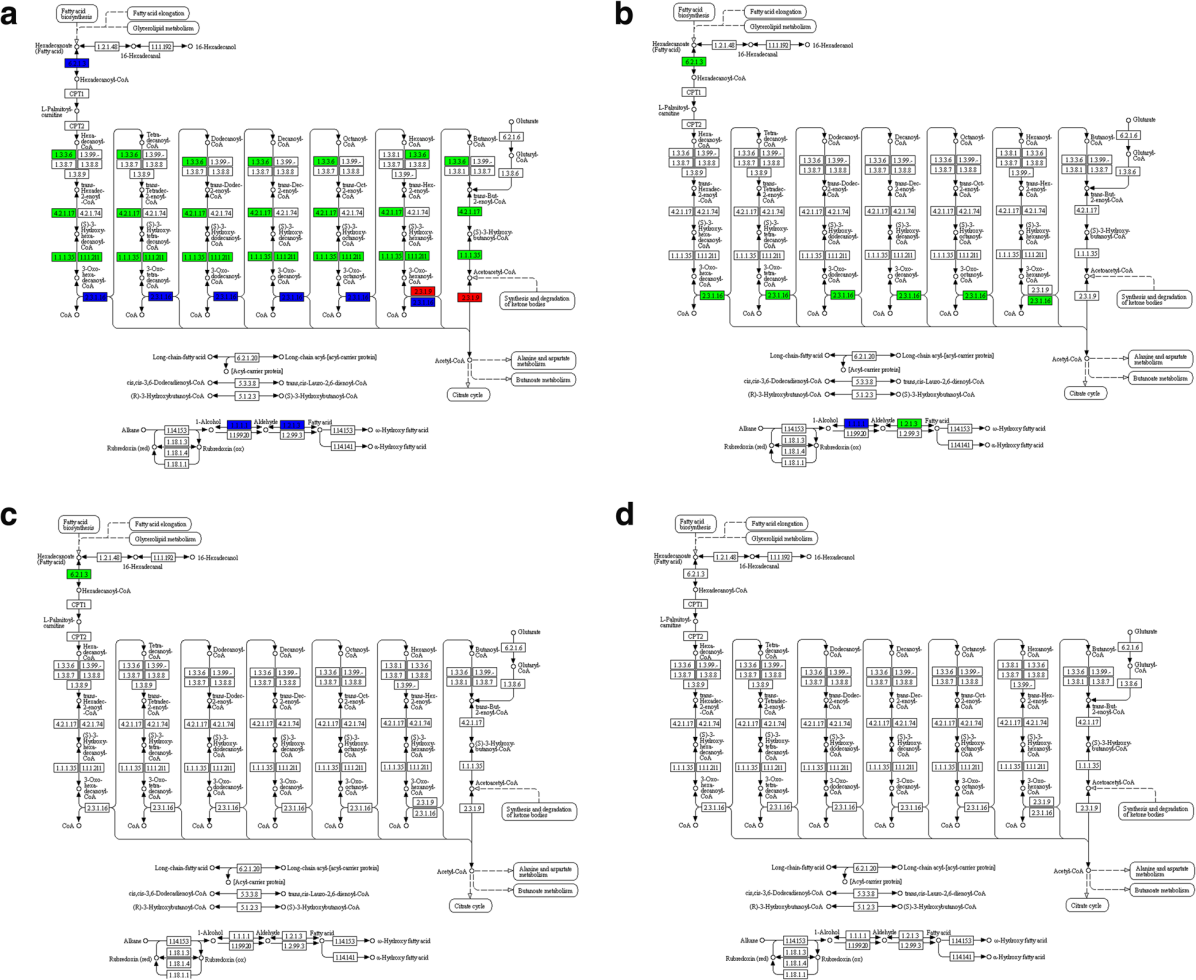
DEG in fatty acid degradation pathway. **a** Comparison L49 vs. H49; **b** Comparison L35 vs. L49; **c,** Comparison L35 vs. H35; and **d** Comparison H35 vs. H49. Red and green denoted up- and down-regulated genes, respectively. Blue denoted the up and down regulated genes both in certain reactions. L indicated Tree L, with low final oil seed content; H indicated Tree H, with high final seed oil content

Fatty acids are degraded in late seed development in order to provide energy during the synthesis of germination enzymes [46]. In KEGG pathway enrichment analysis of DEGs, the fatty acid degradation pathway was enriched in the Comparison L49 vs. H49 (Fig. 2a): many genes in this pathway were highly up regulated in L49 compared to H49 (Fig. 7a). A few genes differed in the Comparison L35 vs. H35 (Fig. 7b) and the Comparison L35 vs. L49 (Fig. 7c), whereas there were no DEGs in the Comparison H35 vs. H49 (Fig. 7d). For instance, the *MULTIFUNCTIONAL family protein* (*MFP2*), *Acyl-coenzyme A oxidase 4* (*ACX4*), *3-ketoacyl-CoA thiolase 2* (*KAT2*), and *Long chain acyl-CoA synthetase 7* (*LACS7*) genes were up-regulated in the L49 samples compared to the others (Fig. 5d). The results indicated that the fatty acid degradation pathway was activated in the late oil accumulation period in the seeds of the Tree L but not in the seeds of Tree H.

### *LEC1*, *LEC1-Like,* and *PII* kept a high expression in the late period of oil accumulation in tree H

**Fig. 8:**
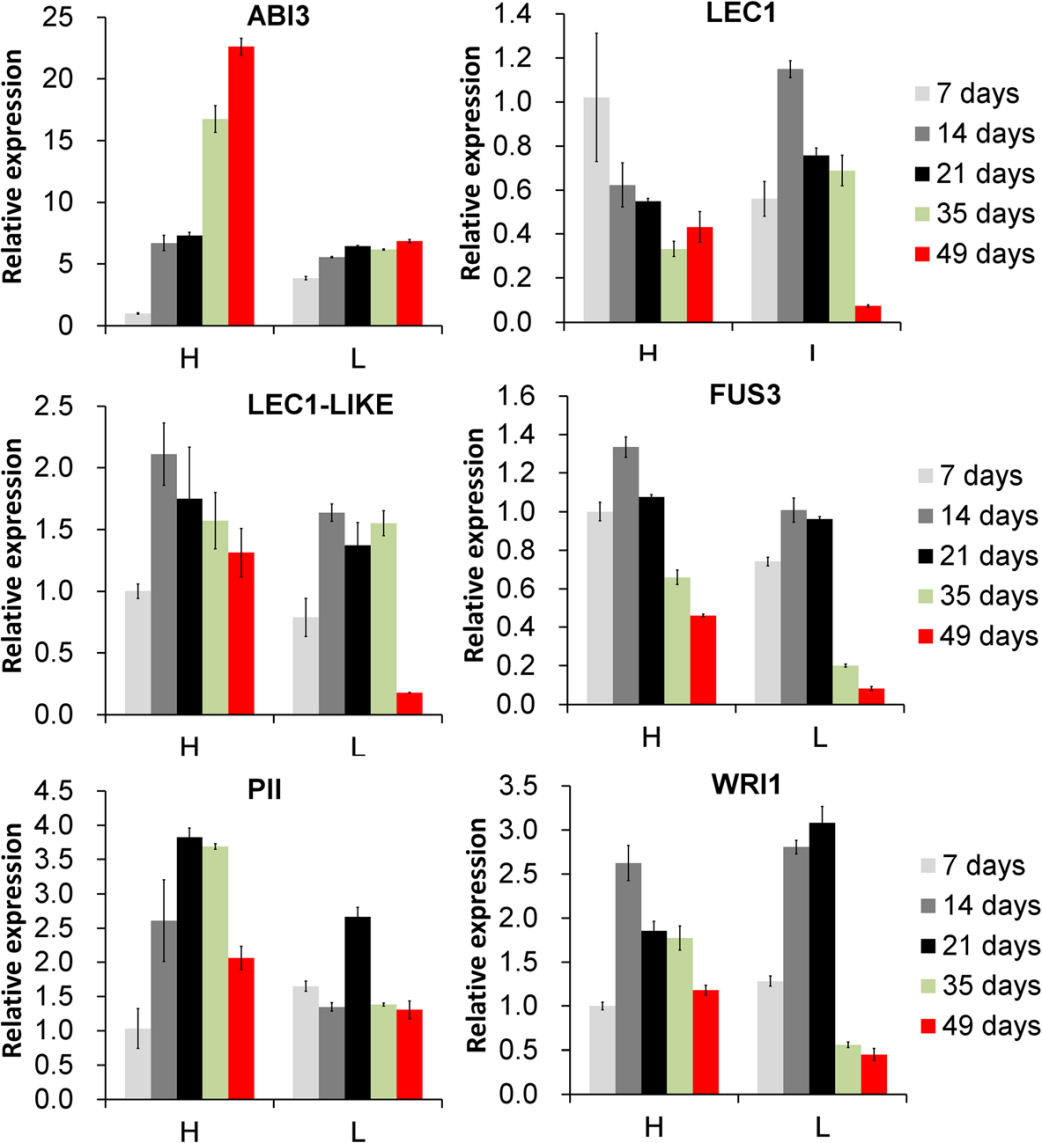
Expression analysis of six transcription factors regulating fatty acid synthesis in tung tree seeds by RT-qPCR. The Sample H7 was as a reference sample that was from the 7^th^ day after the start point of oil accumulation in Tree H. L indicated Tree L, with low final oil seed content; H indicated Tree H, with high final seed oil content. Times in legend indicated time after start point of oil accumulation. Abbreviations: FPKM, reads per kilobase of exon model per million mapped reads (FPKM); ABI3, B3 domain-containing transcription factor ABI3; LEC1, leafy-cotyledon protein 1; LEC1-LIKE, related to LEC1; FUS3, B3 domain-containing transcription factor FUS3; PII, nitrogen regulatory protein P-II homolog; WRII, ethylene-responsive transcription factor WRI1

The expression of plastid *pyruvate kinase* (*PK*), *pyruvate dehydrogenase* (*PDH*), and *acetyl-CoA carboxylase*, the genes encoding key enzymes for fatty acid synthesis in oil accumulation in the seeds, are regulated by *WRI1* [6, 9], a master transcription factor in the regulation of oil accumulation. *WRI1* (Fig. 8) and some of its target genes, for instance, *PK* (Fig. 5a), *PDH* (Additional file 2: Table S5), and CT-α (Additional file 2: Table S5) were quickly up-regulated in the early period of oil accumulation, kept a relatively high expression in the middle period, and declined sharply in the late period in Tree L. This decline was correlated with the end of the oil accumulation, and we consider that these results supported a critical role for *WRI1* in the early and middle period of tung oil accumulation in seeds of Tree L. In Tree H, *WRI1* expression was also up- regulated in the early period of oil accumulation, and kept relatively high expression in the middle period of oil accumulation, but was down regulated in the late periods, similar to the level at 7 days after start point of oil accumulation (Fig. 8). Notably, many genes involved in glycolysis and fatty acid synthesis still had relatively high expression in the late periods of oil accumulation (Fig. 5). For Tree H, *WRI1* is the important TF regulating fatty acid synthesis in the early and middle period of oil accumulation, but may not in the late period. *LEC1* (*leafy-cotyledon protein 1*) [8], *LEC1-Like* (*related to LEC1*) [8] and *PII* (*P-II homolog*) [9], three TFs that also regulate the genes involved in the FA synthesis, were highly expressed in the late period of oil accumulation in Tree H, but were sharply down-regulated in the late period in Tree L (Fig. 8). We interpret this to mean that *PII*, *LEC1*, and *LEC1-Lik*e should play a critical role in the late period of oil accumulation in Tree H. *PII, LEC1-Like,* and *LEC1* were also up-regulated or maintained high expression in the early and middle period of oil accumulation in both trees, which suggests that these three TFs also contributed to the early and middle period of oil accumulation in the two trees. Taking together, the results indicated that oil continual accumulation in the late period should be related to extension of *PII*, *LEC1,* and *LEC1-Like* expression rather than *WRI1* in the late period of oil accumulation.

## Discussion

### Oil accumulation variance and DEGs in the late period of oil accumulation of tree H and L

Tung tree seeds accumulate large amounts of oil during seed development. In this study, maximum endosperm oil content was 55.11% in Tree H and 44.52% in Tree L during the early and middle period of oil accumulation. This was the final oil content reached in seeds of Tree L, but oil accumulation increased subsequently in Tree H until it was about 70%. We speculated that this extended oil accumulation phase is associated with an alternate pathway for high oil content (Additional file 1: Figure S1). DEGs analysis revealed that the global gene expression was quite different in the later period of oil accumulation between the two trees (Additional file 5: Figure S3). Therefore, an alternative molecular mechanism should be responsible for the extension of oil accumulation period that contributed to the high oil content in Tree H.

### Expression pattern for glycolysis and oil biosynthesis genes may account for oil content difference during the early and middle period of oil accumulation between tree H and L

In this study, at the end of the middle accumulation period, oil content of seeds from Tree L (44.52%) was 10.59% less than those from Tree H (55.11%). Many studies that have compared the seed oil content of genotypes reported that high oil content was associated with high expression of genes in the fatty acid and TAG biosynthetic pathway [14–19]. We observed that, in the heatmap, many of the fatty acid and TAG synthesis genes that we studied had generally higher expression during the early and middle period of oil accumulation in Tree H than in Tree L (Fig. 4). However, few genes involved in glycolysis, fatty acid synthesis, TAG assembly, and the TFs regulating oil accumulation were significantly different in Comparisons L7 vs. H7, L14 vs. H14, L21 vs. H21 and L35 vs. H35 [with FDR < 0.01 and ∣log2(fold change)∣ > 1 thresholds (Additional file 2: Table S5)]. The total expression of the four samples in the early and middle period of oil accumulation was calculated for the genes involved in glycolysis, fatty acid synthesis, TAG assembly pathways, and oil-accumulation-related TFs. Statics analysis displayed that the total expression quantity of some critical genes was significantly higher in Tree H than Tree L, specifically that of *Biotin carboxylase of heteromeric ACCase* (*BC*), *Glyceraldehyde-3-phosphate dehydrogenase* (*GAPCP2*), *Plastidial pyruvate kinase 2* (*PK2*) and *Putative glucose-6-phosphate 1-epimerase* (*PEG1–1*) (Additional file 7: Figure S5). Thus, the higher total expression of genes involved in glycolysis, fatty acid, and TAG assembly in Tree H seeds may be responsible for the oil content difference of 10.59% between the two trees during the early and middle period of oil accumulation.

### High expression persistent of genes involved in glycolysis and oil biosynthesis during the late period of oil accumulation may contribute to the increase in oil content during the late accumulation phase of tree H

The oil accumulation phase was extended in Tree H, beyond that of Tree L. This extra three-week accumulation time resulted in a total of 13.85% more oil in Tree H than in Tree L (Fig. 1). The glycolysis pathway was enriched in two comparisons: L35 vs. L49 and L49 vs. H49, which lead us to suggest that the glycolysis pathway was important to oil accumulation during the late period. Expression patterns for most DEGs involved in the conversion of pyruvate (Figs. 4a and 5a) to common fatty acids (Figs. 4b and 5b), α-ESA production and TAG assembly (Figs. 4c and 5c) were similar, all with a up regulation or high expression in the early and middle period for the two trees, and the high expression persisted into the late period of tung oil accumulation, in Tree H but not in Tree L. Thus we concluded that oil quick accumulation period extension was closely related to the persistence of the relatively high expression of genes involved in glycolysis, fatty acid synthesis, α-ESA accumulation, and TAG assembly in the late period of oil accumulation, finally leading to high oil content in Tree H. Besides, these data also indicated that, during the tung oil rapid accumulation stage, the coordination of genes was not only between fatty acid synthesis and glycolysis genes [20], but also to those genes involved in α-ESA production and TAG assembly.

### Alternative TFs should regulate the extension of fatty acid synthesis genes expression during the late period of oil accumulation in tree H

*WRI1* controls oil accumulation in seeds by directly regulating expression of many genes involved in fatty acid synthesis [4, 7, 10, 42, 47]. Based on the expression profile of *WRI1* in our study, however, it should be important in the early and middle period of tung oil accumulation in Tree L and Tree H, as reported for Arabidopsis [4]. *WRI1* is reported that no more improved oil accumulation in the late period of *Arabidopsis* seed development [4]. In this study, some alternative TFs may be take on the role of *WRI1* in seeds of Tree H, extending high expression of the *WRI1*-target genes into the late period of oil accumulation. In support of this suggestion, we observed high expression of three oil-accumulation-related TFs: *Nitrogen regulatory protein P-II homolog* (*PII*) [9], *Leafy cotyledon protein 1 (LEC1*) [8], *and related to LEC1 (LEC1-Like*) [8] during the late period of oil accumulation in Tree H. *Abscisic acid-insensitive3* (*ABI3*) [48–50] and *FUSCA3 (FUS)* [3, 51] are two TFs that regulate seed maturation. It has been demonstrated recently, by To et al. [50], that *ABI3* and *FUS3* participated in a highly redundant gene regulation network that controls most aspects of seed maturation, indicating a probable role in oil accumulation. *FUS3* shared similar expression pattern as *WRI1* for the two trees. ABI3 were noted to be largely up regulated in the late oil accumulation phase in Sample H49, but not in L49 (Fig. 8). Thus, *ABI3* may participate in an alternative molecular mechanism that promoted oil accumulation in the late period of oil accumulation in Tree H. We must note, however, that the aforementioned four TFs that may play a critical role in the late period of oil accumulation were largely up-regulated in the early and middle period of oil accumulation of both trees, which supported the suggestion that other different expression TFs should take an important role in suppressing the high expression of the four TFs in the late period of oil accumulation in the seeds of Tree L.

To search for the candidates TFs, we identified 60 differentially expressed TFs (with expression levels exceeding 20 FPKM for at least one of samples during the 20 samples) from the 893 DEGs that were both significantly different in Comparisons L35 vs. L49 and L49 vs. H49, but that were not common between Comparisons H35 vs. H49 and L35 vs. H35 (Fig. 4d, Additional file 2: Table S5). Additional file 8: Figure S6 showed that several TFs were largely up regulated in L49 but had little or no expression in all other samples. These TFs included three *WRKY* TFs, three *MYB* TFs, as well as some others. Little is known about the function of these TFs in oil accumulation [52, 53], and further investigation of their role in seed development would be investigated.

**Fig. 9:**
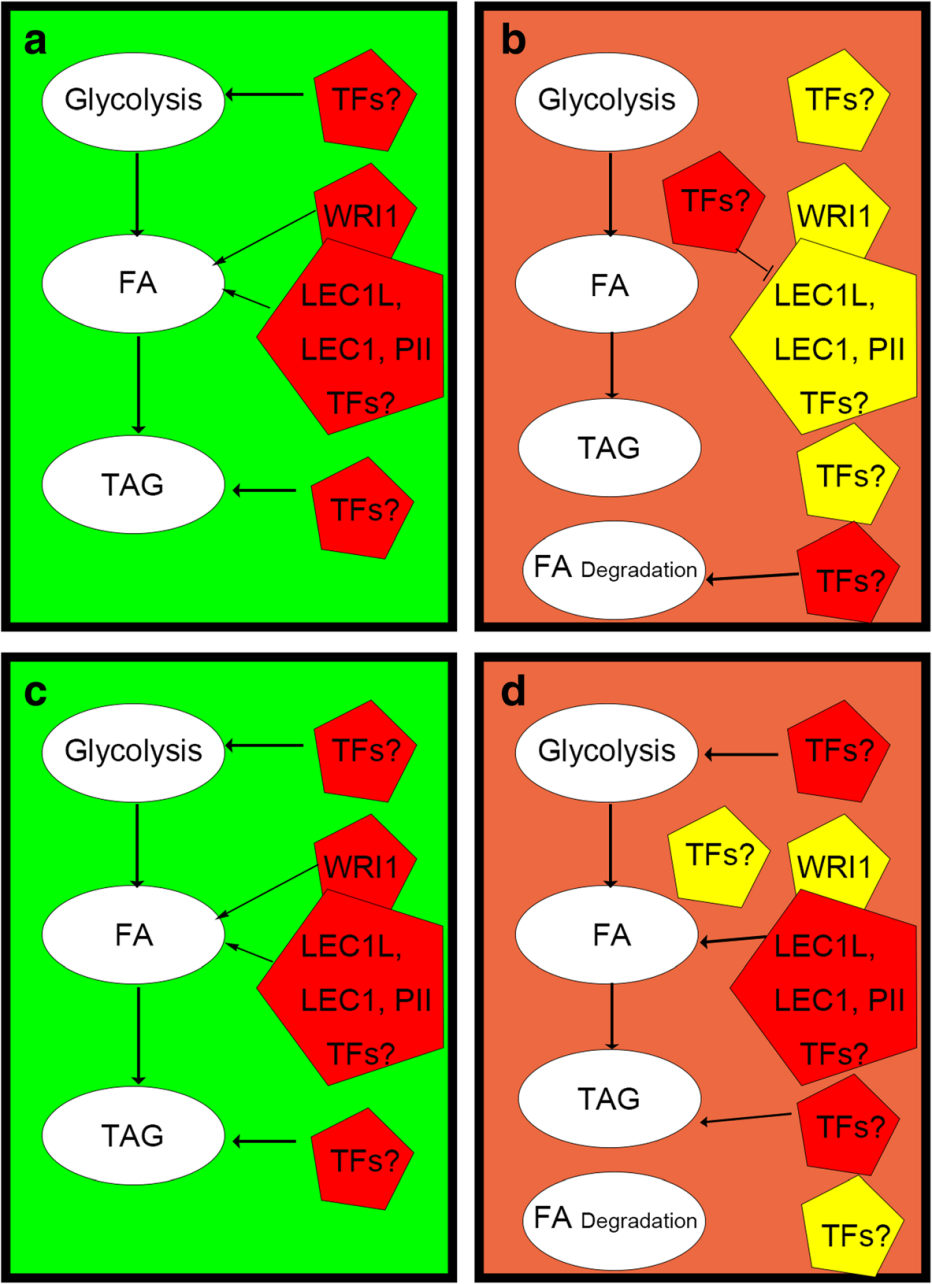
The proposed regulation mode for seed oil accumulation in Tree H and L. **a** and **b** Gene expression in Tree L, with low final oil content; **a** Early and middle period of oil accumulation; **b** Late period of oil accumulation. **c** and **d** Gene expression in Tree H, with high final oil content; **c** Early and middle period of oil accumulation; **d** Late period of oil accumulation. Red and yellow in the pentagon indicated high and low expression, respectively. (FA, fatty acids; TAG, tri-acyl glycerides; TF, transcription factors; LEC1L, LEC1-LIKE)

Finally, based on the results, we propose a regulation mode, illustrated in Fig. 9 that may depict the molecular mechanism for the different oil contents in the two trees we have studied.

## Conclusions

This study indicated that continued accumulation of oil in the late period could lead to high oil content, which is associated with relatively high expression of the relevant genes in glycolysis, fatty acid synthesis and tri-acyl glyceride assembly during the late period of oil accumulation. *PII*, *LEC1,* and *LEC1-LIKE* rather than *WRI1* should play an important role in oil continual accumulation in the late period. This study provided novel insight into the variation in seed oil content and informed plant breeding strategies to maximize oil yield.

## Additional files

Additional file 1: **Figure S1.** Varied oil accumulation period in tung tree seeds. **a**, Four accessions with about 50% of oil content; **b**, Four accessions with about 60% oil content; **c**, Four accessions with about 70% oil content. The Arab number in the x-axis indicated sampling date (month and day; for example, 713 is July 13). We defined the period with the apparent increase for oil content in tung tree seed as rapid oil accumulation. Total oil quick accumulation day was calculated and marked in the bottom right corner. (TIF 1717 kb)

Additional file 2: **Table S6.** The nucleotide sequences of real-time PCR primers. **Table S1.** A summary of sequence assembly after illumine sequencing. L indicated Tree L, with low final oil seed content; H indicated Tree H, with high final seed oil content; these were followed by a number that indicated the days after the start point of oil accumulation, the number after the dash indicates replicates. **Table S2.** A summary of assembled reads blasting to the tung tree genomic sequences. **Table S3. ** A summary of differentially expressed genes (DEGs). L indicated Tree L, with low final oil seed content; H indicated Tree H, with high final seed oil content; these were followed by a number that indicated the days after the start point of oil accumulation. **Table S4.** KEGG enrichments analysis for seven comparisons. Comparisons were: H35 vs. H49, L35 vs. L49, L7 vs. H7, L14 vs. H14, L21 vs. H21, L35 vs. H35, L49 vs. H49. (L indicated Tree L, with low final oil seed content; H indicated Tree H, with high final seed oil content; these were followed by a number that indicated the days after the start point of oil accumulation.). **Table S5.** A summary of DEGs related to oil accumulation detected in this study. (XLSX 4911 kb)

Additional file 3: **Figure S7.** Transcript expression of 60s ribosomal protein L18a (Rp119A, Tung tree ID tung.gene.scaffold67.00040) in the ten samples of tung tree seeds. (TIFF 4825 kb)

Additional file 4: **Figure S2.** Differentially expressed genes (DEGs) number for seven comparisons. L indicated Tree L, with low final oil seed content; H indicated Tree H, with high final seed oil content; these were followed the number of days after the start point of oil accumulation. (TIF 2070 kb)

Additional file 5: **Figure S3.** Hierarchical clustering of all the DEGs. L indicated Tree L, with low final oil seed content; H indicated Tree H, with high final seed oil content; these were followed the number of days after the start point of oil accumulation; the number after the dash indicates replicates. (TIF 635 kb)

Additional file 6: **Figure S4.** Transcript expression of 12 DEGs related to acyl metabolism. These DEGs were remarkably different expression in the L49 samples (from Tree L late in oil accumulation) compared to the other samples. L indicated Tree L, with low final oil seed content; H indicated Tree H, with high final seed oil content. Times in legend indicated days after start point of oil accumulation. Abbreviations: FPKM, reads per kilobase of exon model per million mapped reads; LASC8, long chain acyl-CoA synthetase 8; KCS4, 3-ketoacyl-CoA synthase 4; EK, probable ethanolamine kinase; CK, probable choline kinase 2; PP, putative lipid phosphate phosphatase; LPP2, lipid phosphate phosphatase 2; LTP5, non-specific lipid-transfer protein D; EARLI 1, lipid transfer protein; CSD1, phosphatidate cytidylyltransferase (TIF 7139 kb)

Additional file 7: **Figure S5.** Total gene expression quantity during the early and middle period of tung oil accumulation. L indicated Tree L, with low final oil seed content; H indicated Tree H, with high final seed oil content; total gene expression values were all significantly different between the two trees according to Student’s t test, *P* < 0.05 (Additional file 1: Table S5). Abbreviations: FPKM, reads per kilobase of exon model per million mapped reads (FPKM); BC, Biotin carboxylase of heteromeric ACCase; GAPCP2, Glyceraldehyde-3-phosphate dehydrogenase; PK2, Plastidial pyruvate kinase 2; PEG1–1, Putative glucose-6-phosphate 1-epimerase. (TIFF 1092 kb)

Additional file 8: **Figure S6.** Transcript expression of 12 transcription factors with remarkably different expression in Samples L49. L indicated Tree L, with low final oil seed content; H indicated Tree H, with high final seed oil content; times in legend indicated days after start point of oil accumulation. Abbreviations: FPKM, reads per kilobase of exon model per million mapped reads (FPKM), WRKY1, *WRKY transcription factor 1*; WRKY41, *WRKY transcription factor 41*; WRKY07, *WRKY transcription factor 07*; MYB1R1, *MYB1R1 transcription factor*; MYB44, *MYB44 transcription factor*; MYB108, *MYB108 transcription factor*; ERF9, *Ethylene-responsive transcription factor 9*; ERF112, *Ethylene-responsive transcription factor ERF112*; AFC2, *Serine/threonine-protein kinase AFC2*; LRR-RLK1, *Probable LRR receptor-like serine/threonine-protein kinase*; H2A.1, *Probable histone H2A.1*; WER, *Transcription factor WER* (TIFF 10090 kb)

## Abbreviations

AB13: B3 domain-containing transcription factor ABI3
ACX4: Acyl-coenzyme A oxidase 4
ADH: Alcohol dehydrogenase 1
AFC2: Serine/threonine-protein kinase AFC2
ALDH2: Aldehyde dehydrogenase family 2
ALDH7: Aldehyde dehydrogenase family 7
BC: Biotin carboxylase of heteromeric ACCase
CK: Probable choline kinase 2
CSD1: Phosphatidate cytidylyltransferase
DGAT2: Type 2 diacylglycerol acyltransferase
EARLI 1: Lipid transfer protein
EK: Probable ethanolamine kinase
ENO2: Enolase
ENR: Enoyl-ACP reductase
ERF112: Ethylene-responsive transcription factor ERF112
ERF9: Ethylene-responsive transcription factor 9
FADX: Fatty acid conjugase/desaturase
FPKM: Reads per kilobase of exon model per million mapped reads (FPKM)
FUS3: B3 domain-containing transcription factor FUS3
GAPCP2: Glyceraldehyde-3-phosphate dehydrogenase
GPDH: Glycerol-3-phosphate dehydrogenase
H2A.1: Probable histone H2A.1
i-PGM: 2,3-bisphosphoglycerate-independent phosphoglycerate mutase
KAR: Ketoacyl-ACP reductase
KAS I: Ketoacyl-ACP synthase I
KAT2: 3-ketoacyl-CoA thiolase 2
KCS4: 3-ketoacyl-CoA synthase 4
LACS7: Long chain acyl-CoA synthetase 7
LASC8: Long chain acyl-CoA synthetase 8
LASC9: Long chain acyl-CoA synthetase 9
LEC1: Leafy-cotyledon protein 1
LEC1-LIKE: related to LEC1
LPAA2: 1-acyl-sn-glycerol-3-phosphate acyltransferase 2
LPP2: Lipid phosphate phosphatase 2
LRR-RLK1: Probable LRR receptor-like serine/threonine-protein kinase
LTP5: Non-specific lipid-transfer protein D
MFP2: Multifunctional family protein
MYB108: MYB108 transcription factor
MYB1R1: MYB1R1 transcription factor
MYB44: MYB44 transcription factor
PCK: Phosphoenolpyruvate carboxykinase
PEG1–1: Putative glucose-6-phosphate 1-epimerase
PEG-2: Putative glucose-6-phosphate 1-epimerase
PII: Nitrogen regulatory protein P-II homolog
PK2: Plastidial pyruvate kinase 2
PK2: Plastidial pyruvate kinase 2
PP: Putative lipid phosphate phosphatase
WER: Transcription factor WER
WRII: Ethylene-responsive transcription factor WRI1
WRKY07: WRKY transcription factor 07
WRKY1: WRKY transcription factor 1
WRKY41: WRKY transcription factor 41
