# Supplementary figures and images for: Molecular mechanism of the extended oil accumulation phase contributing to the high seed oil content for the genotype of tung tree (Vernicia fordii)
Source: BMC Plant Biol. 2018 Oct 19;18:248. doi: 10.1186/s12870-018-1458-3 (PMC6195728; doi:10.1186/s12870-018-1458-3)

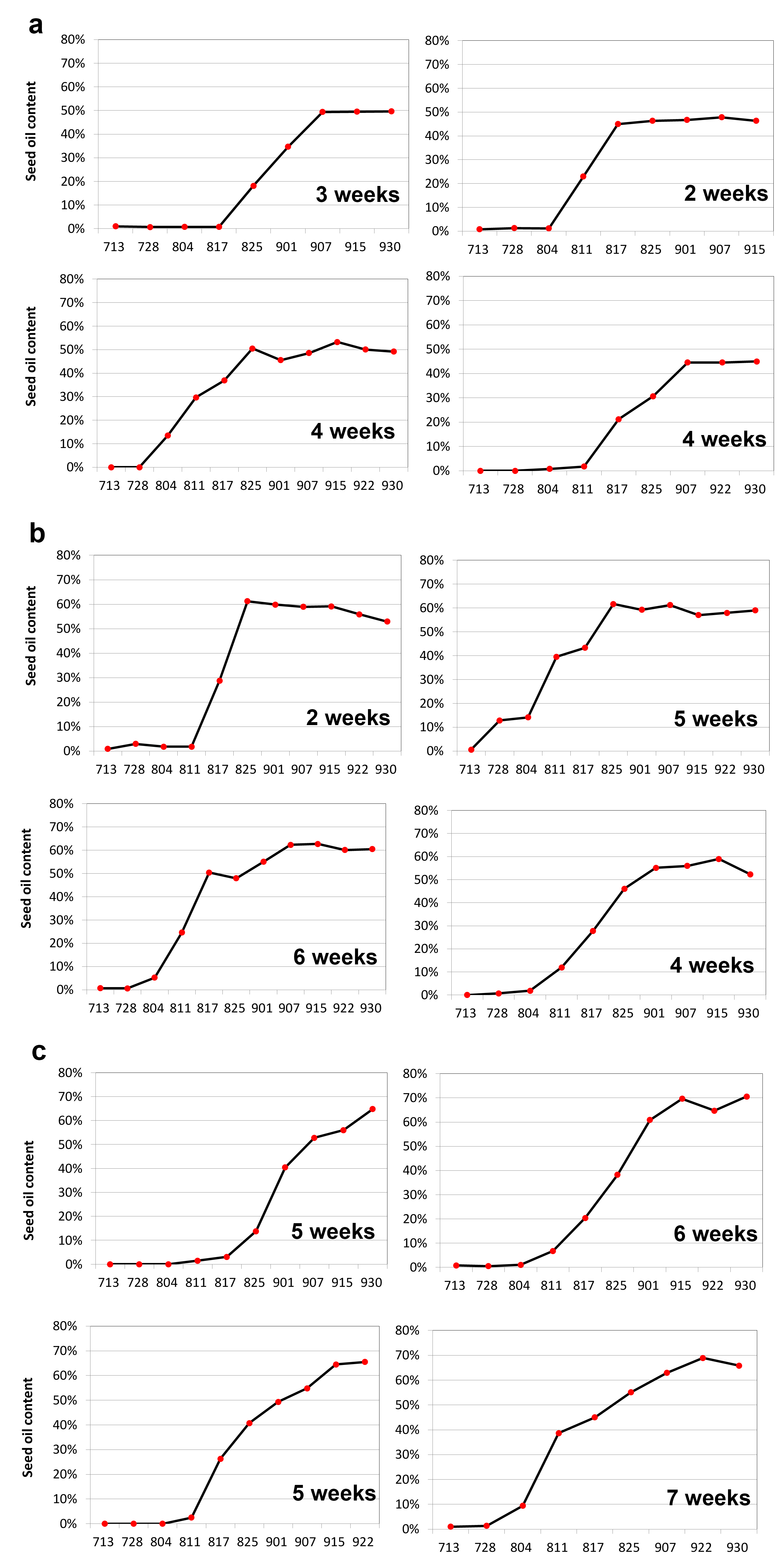

Supplement: Supplementary file 1 — Figure S1. Varied oil accumulation period in tung tree seeds. a, Four accessions with about 50% of oil content; b, Four accessions with about 60% oil content; c, Four accessions with about 70% oil content. The Arab number in the x-axis indicated sampling date (month and day; for example, 713 is July 13). We defined the period with the apparent increase for oil content in tung tree seed as rapid oil accumulation. Total oil quick accumulation day was calculated and marked in the bottom right corner. (TIF 1717 kb) [file 12870_2018_1458_MOESM1_ESM.tif]

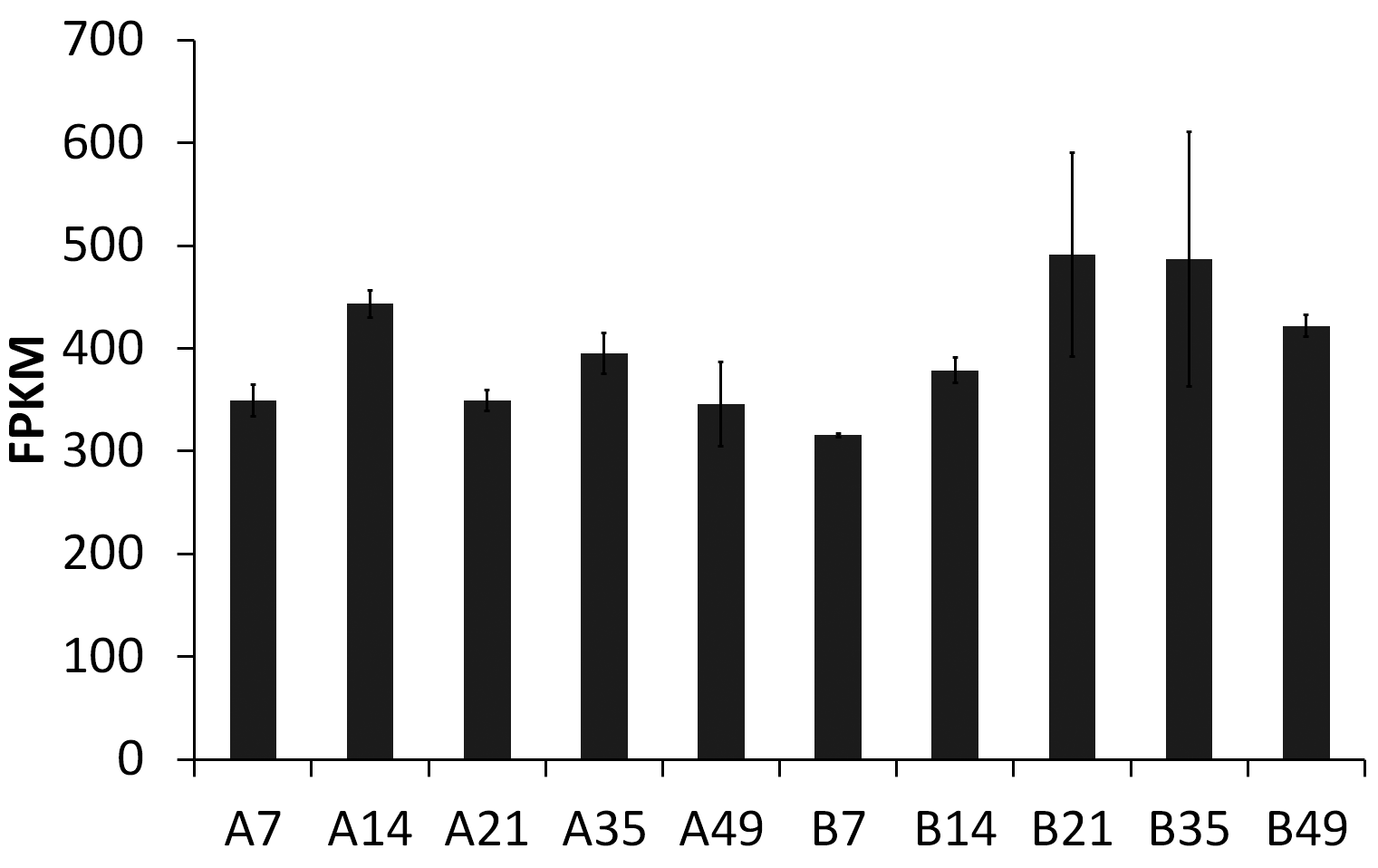

Supplement: Supplementary file 3 — Figure S7. Transcript expression of 60s ribosomal protein L18a (Rp119A, Tung tree ID tung.gene.scaffold67.00040) in the ten samples of tung tree seeds. (TIFF 4825 kb) [file 12870_2018_1458_MOESM3_ESM.tiff]

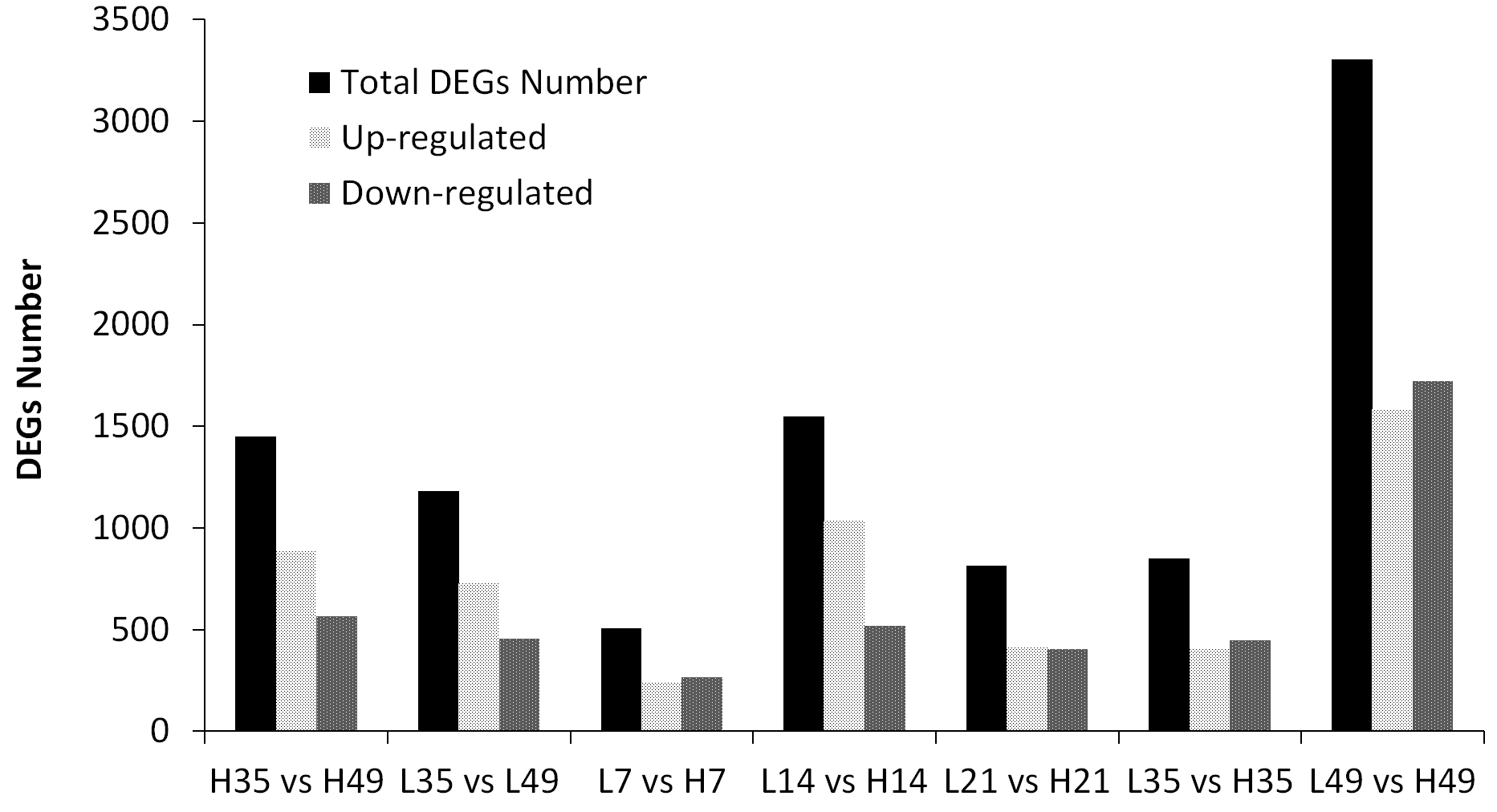

Supplement: Supplementary file 4 — Figure S2. Differentially expressed genes (DEGs) number for seven comparisons. L indicated Tree L, with low final oil seed content; H indicated Tree H, with high final seed oil content; these were followed the number of days after the start point of oil accumulation. (TIF 2070 kb) [file 12870_2018_1458_MOESM4_ESM.tif]

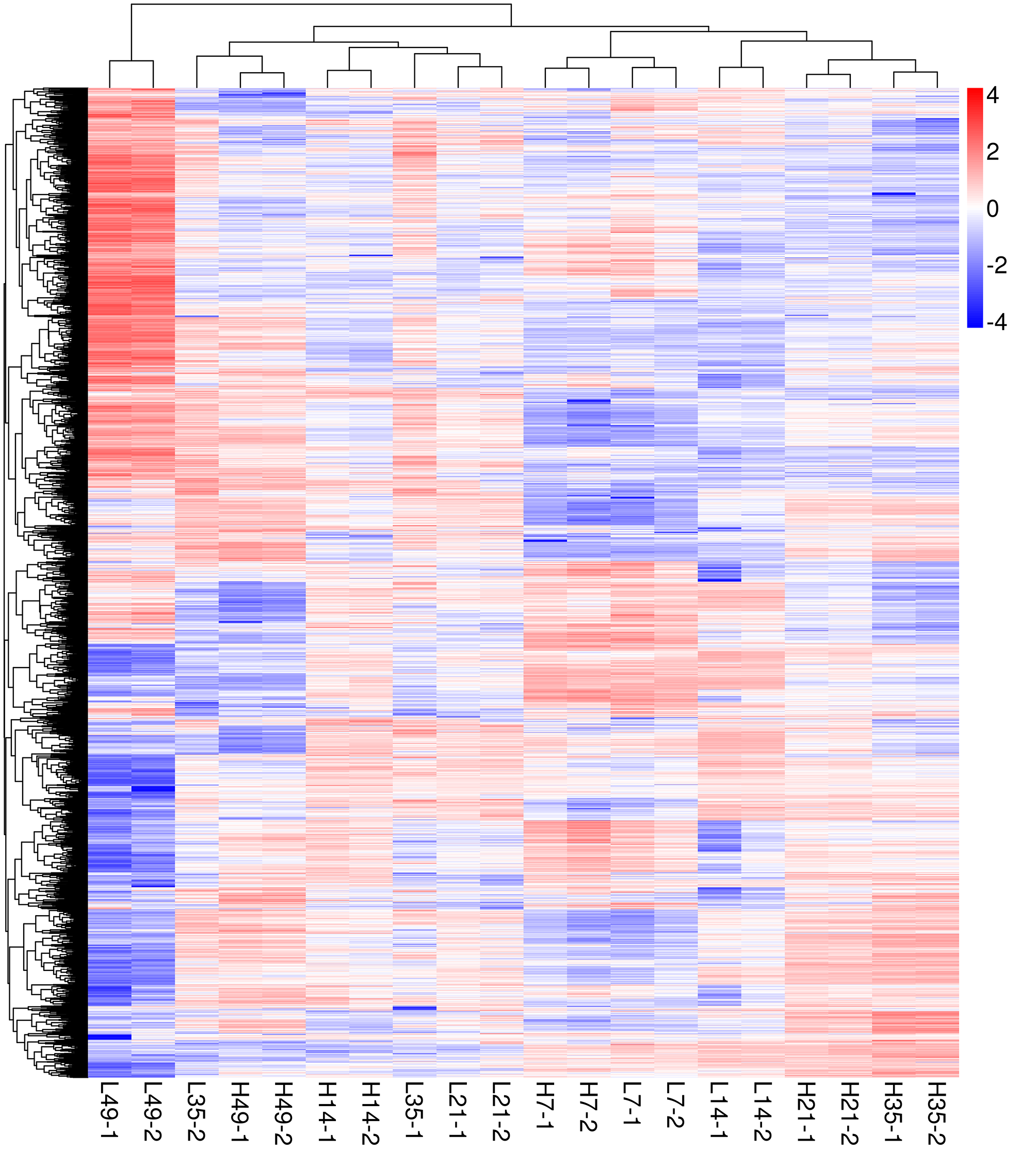

Supplement: Supplementary file 5 — Figure S3. Hierarchical clustering of all the DEGs. L indicated Tree L, with low final oil seed content; H indicated Tree H, with high final seed oil content; these were followed the number of days after the start point of oil accumulation; the number after the dash indicates replicates. (TIF 635 kb) [file 12870_2018_1458_MOESM5_ESM.tif]

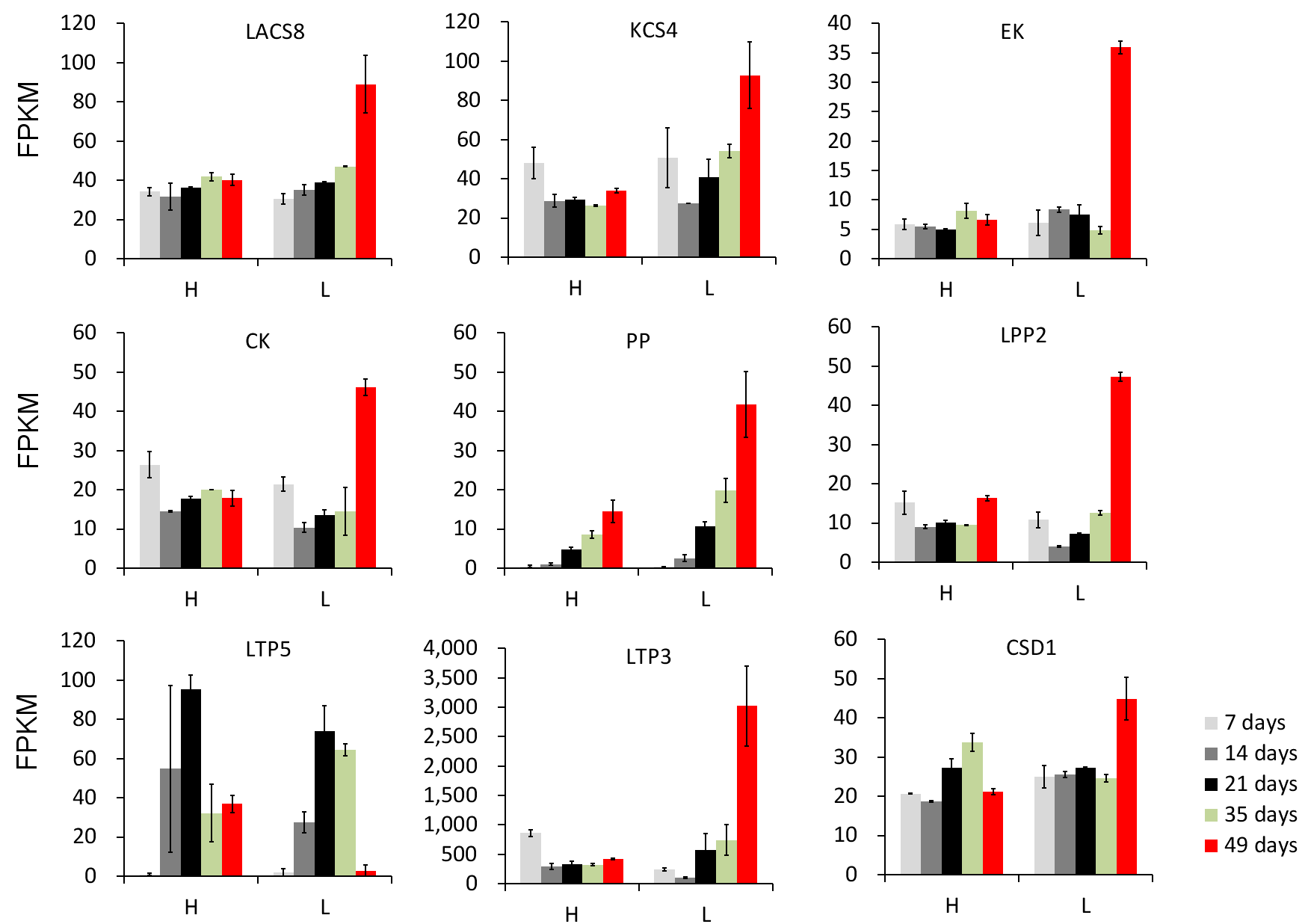

Supplement: Supplementary file 6 — Figure S4. Transcript expression of 12 DEGs related to acyl metabolism. These DEGs were remarkably different expression in the L49 samples (from Tree L late in oil accumulation) compared to the other samples. L indicated Tree L, with low final oil seed content; H indicated Tree H, with high final seed oil content. Times in legend indicated days after start point of oil accumulation. Abbreviations: FPKM, reads per kilobase of exon model per million mapped reads; LASC8, long chain acyl-CoA synthetase 8; KCS4, 3-ketoacyl-CoA synthase 4; EK, probable ethanolamine kinase; CK, probable choline kinase 2; PP, putative lipid phosphate phosphatase; LPP2, lipid phosphate phosphatase 2; LTP5, non-specific lipid-transfer protein D; EARLI 1, lipid transfer protein; CSD1, phosphatidate cytidylyltransferase (TIF 7139 kb) [file 12870_2018_1458_MOESM6_ESM.tif]

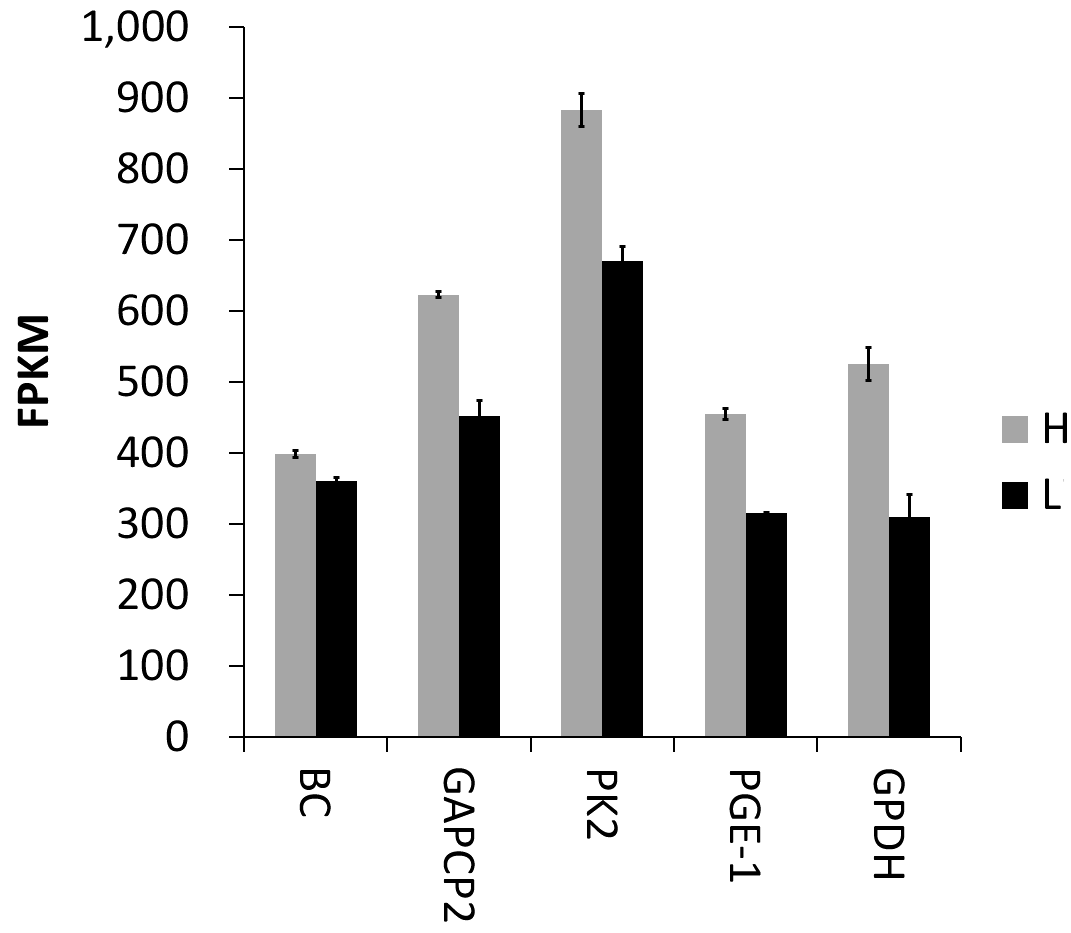

Supplement: Supplementary file 7 — Figure S5. Total gene expression quantity during the early and middle period of tung oil accumulation. L indicated Tree L, with low final oil seed content; H indicated Tree H, with high final seed oil content; total gene expression values were all significantly different between the two trees according to Student’s t test, P < 0.05 (Additional file 1: Table S5). Abbreviations: FPKM, reads per kilobase of exon model per million mapped reads (FPKM); BC, Biotin carboxylase of heteromeric ACCase; GAPCP2, Glyceraldehyde-3-phosphate dehydrogenase; PK2, Plastidial pyruvate kinase 2; PEG1–1, Putative glucose-6-phosphate 1-epimerase. (TIFF 1092 kb) [file 12870_2018_1458_MOESM7_ESM.tiff]

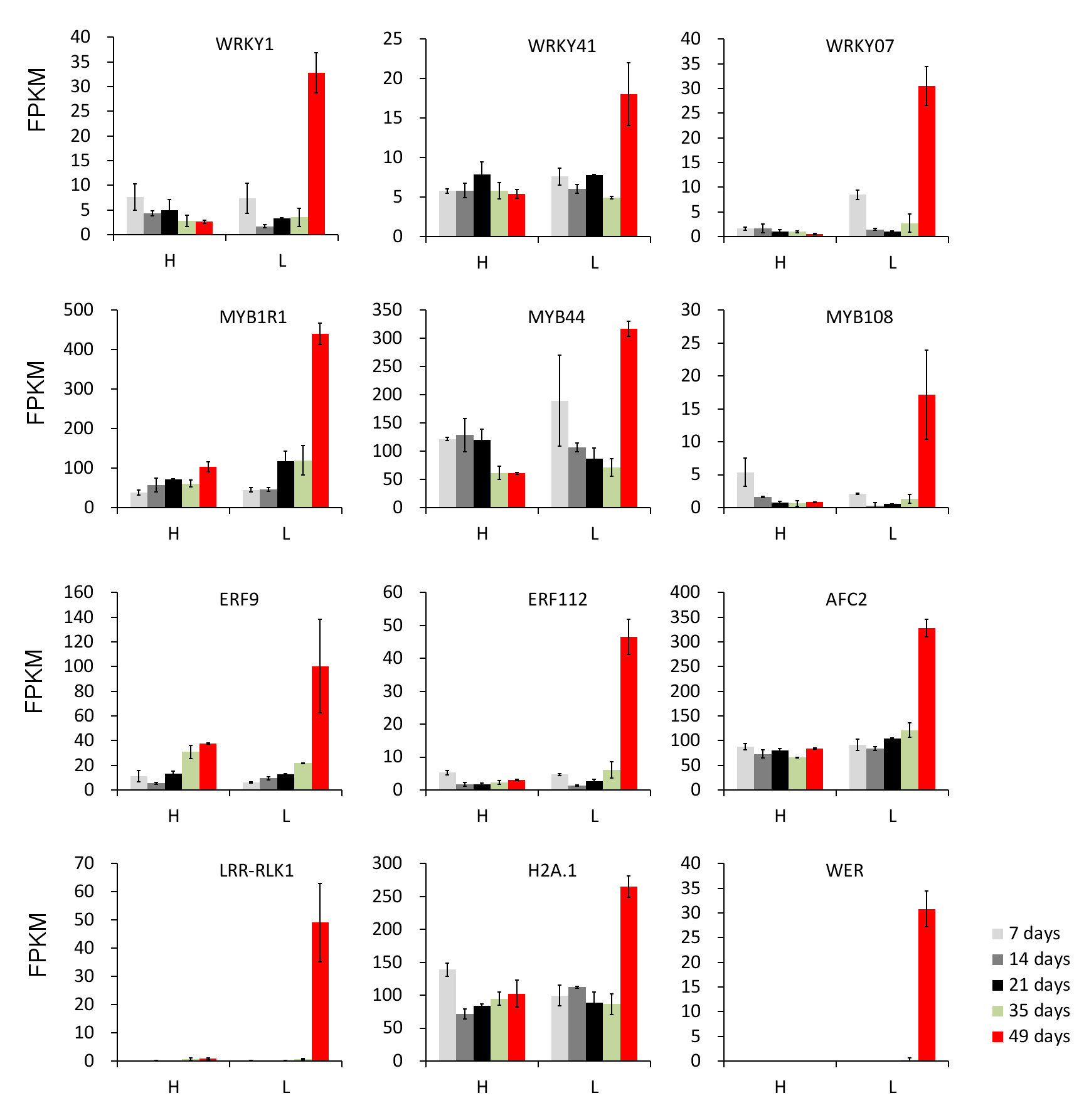

Supplement: Supplementary file 8 — Figure S6. Transcript expression of 12 transcription factors with remarkably different expression in Samples L49. L indicated Tree L, with low final oil seed content; H indicated Tree H, with high final seed oil content; times in legend indicated days after start point of oil accumulation. Abbreviations: FPKM, reads per kilobase of exon model per million mapped reads (FPKM), WRKY1, WRKY transcription factor 1; WRKY41, WRKY transcription factor 41; WRKY07, WRKY transcription factor 07; MYB1R1, MYB1R1 transcription factor; MYB44, MYB44 transcription factor; MYB108, MYB108 transcription factor; ERF9, Ethylene-responsive transcription factor 9; ERF112, Ethylene-responsive transcription factor ERF112; AFC2, Serine/threonine-protein kinase AFC2; LRR-RLK1, Probable LRR receptor-like serine/threonine-protein kinase; H2A.1, Probable histone H2A.1; WER, Transcription factor WER (TIFF 10090 kb) [file 12870_2018_1458_MOESM8_ESM.tiff]
